# Accounting for Variability Across Years, Depths, and Non‐Target Reads Improves Quantification of Root Community Composition in Plant Mixtures

**DOI:** 10.1111/1755-0998.70214

**Published:** 2026-10-16

**Authors:** Emma K. Rice, Jason C. Keagy, Terrence H. Bell, Liana T. Burghardt, Jason P. Kaye, Carolyn J. Lowry

**Affiliations:** ^1^ Intercollege Graduate Degree Program in Ecology The Pennsylvania State University University Park Pennsylvania USA; ^2^ Department of Plant Science The Pennsylvania State University University Park Pennsylvania USA; ^3^ Department of Ecosystem Science and Management The Pennsylvania State University University Park Pennsylvania USA; ^4^ Department of Physical and Environmental Sciences University of Toronto Scarborough Toronto Ontario Canada

**Keywords:** calibration mixtures, cover crops, metabarcoding, plant communities, root abundance

## Abstract

One key challenge in characterizing plant communities is determining the relative abundance of distinct species in root biomass. Metabarcoding is increasingly used to estimate a species' proportion in root mass based on its proportion in sample reads, but results have been mixed in accurately converting sequencing output to species mass in mixture. We created calibration mixtures from four focal plant species to determine how the association between a species' proportion in mass and its proportion in sequencing reads of the *rbcL* chloroplast gene varied across sample years, soil depth, and non‐target proportion of reads. We also determined whether including these explanatory variables in prediction models could improve accuracy. We found the association between a species' proportion in reads and its proportion in mass varied across years and non‐target proportion of reads, and to a lesser extent, rooting depth, and incorporating these explanatory factors into prediction models improved prediction accuracy. A secondary objective was to determine the number of calibration levels and sequencing depth required to accurately predict a species' proportion in mass. Through simulations, we estimated that 42 calibration samples (seven proportional levels * three years * two depths) and 700 reads per sample consistently obtained accurate predictions for simple mixtures. The association between a species' proportion in mass and proportion in reads can vary considerably, and our study suggests that measuring and explaining this variation can lead to more accurate predictions of the relative abundance of plants within root biomass using metabarcoding.

## 1 Introduction

Roots are integral to understanding how plants are interacting with one another and their belowground environment (Belter and Cahill 2015; Casper and Jackson 1997; De Kroon et al. 2012; Homulle et al. 2022; Mommer et al. 2016; Tilman 1988; Weiss et al. 2019). Yet historically, it was nearly impossible to quantify the relative abundance of roots from distinct plant species growing in diverse communities. Root fragments are both difficult to visually identify to species and time‐intensive to excavate. When studying the composition of diverse plant mixtures, researchers most often quantify the aboveground biomass of individual species and use this measure as a proxy for the relative abundance of the whole plant (above and belowground). However, it is unlikely that aboveground community composition can accurately predict belowground root composition since plant species vary in how they adjust biomass allocation to roots versus shoots in response to both environmental variation and the neighbouring community (Poorter et al. 2012). Roots provide many ecosystem services that are essential for both soil health and conservation, including nutrient retention, biological nitrogen fixation and reduced soil compaction and erosion (Bardgett and Van Der Putten 2014; Blesh et al. 2019; Freschet et al. 2021). Understanding the relative abundance of individual species in belowground biomass can help the design of diverse plant communities that are better at provisioning root‐linked ecosystem services.

In the near‐term, accurate assessments of underground root composition will need to rely on non‐visual markers, such as DNA sequence composition. As next‐generation sequencing becomes less expensive to apply, it is an increasingly viable option for estimating both the presence and abundance of distinct plant species in a community's root biomass. Metabarcoding involves amplifying a specific gene fragment using primers that can target a range of organisms to detect sequence variants from across a set of environmental samples (Liu et al. 2021; Zhou et al. 2011). Metabarcoding in plants often targets plastid genes (e.g., *rbcL*, *trnL*, *matK*), the internal transcribed spacer of nuclear ribosomal DNA (ITS; typically ITS2) and the *trnH‐psbA* intergenic spacer (Bell et al. 2017; Chen et al. 2010; De Boer 2022; Hollingsworth 2011; Hollingsworth et al. 2016; Kress and Erickson 2007; Mahadani et al. 2022). In previous studies, the *rbcL* chloroplast gene exhibited high universality but variable species discrimination, identifying between 26% to 70% of species and *matK*, another popular plastid plant marker, differentiated between 45% to 66% of species (CBOL Plant Working Group et al. 2009; China Plant BOL Group et al. 2011; Kress and Erickson 2007). ITS2, a short marker within the larger ITS region, has high taxonomic resolution, identifying 55% to 86% of species (China Plant BOL Group et al. 2011; Moorhouse‐Gann et al. 2018). Often promoted as a secondary marker to accompany plastid barcodes, ITS2 can have issues differentiating between closely related taxa and with universal amplification (De Boer 2022). Unlike in animal and microbial research, where specific markers have been well established, plant research offers several recommended options, with the most suitable choice often depending on the species of interest.

Broadly, metabarcoding has been used to identify diet preferences in herbivores (Bell et al. 2021; Guo et al. 2018; Nakahama et al. 2021; Wen et al. 2024; Williams et al. 2021) and omnivores (Schumm et al. 2023), pollinator preferences (Bell et al. 2019; Pornon et al. 2016), and is increasingly used to investigate root communities (Hassan et al. 2019; Hiiesalu et al. 2012; Lamb et al. 2016; Matesanz et al. 2019), especially when designing plant species‐specific primers is not efficient or feasible (Wagemaker et al. 2020). Studies on root mixtures have predominantly focused on species richness (Hiiesalu et al. 2012; Illuminati et al. 2021; Isola et al. 2021; Jones et al. 2011; Lamb et al. 2016; Rucińska et al. 2022), but a few studies have applied metabarcoding to quantify the relative abundance of specific plant communities, with varying success (Hassan et al. 2019; Li et al. 2018; Matesanz et al. 2019). Despite efforts to improve estimation of species‐specific abundance from sequencing output (Matesanz et al. 2019; McLaren et al. 2019; Thomas et al. 2016; Zhu et al. 2023), there is some debate on whether this approach is appropriate for moving beyond organism presence to relative abundance (Barlow et al. 2020; Deagle et al. 2019; Fonseca 2018; Nichols et al. 2018), because it is challenging to accurately convert number of species reads to predicted species mass. For example, Matesanz et al. (2019) investigated belowground abundance in Mediterranean shrubland and found that two of the five focal species were either over‐ or underestimated.

A major challenge with using metabarcoding to accurately quantify species relative abundance is that bias is introduced throughout the experimental pipeline (McLaren et al. 2019), leading to differences between the measured and true relative abundance of species. For example, ITS2 can have paralogous gene copies and amplification of fungal DNA, potentially confounding the interpretation of plant species relative abundance (China Plant BOL Group et al. 2011; Hollingsworth 2011; Moorhouse‐Gann et al. 2018). Additionally, the copies of individual plastid genes can vary across cells and plant tissues (CBOL Plant Working Group 2009). Bias can occur from differences in species gene copy or plastid number, unequal DNA extraction, PCR amplification, DNA sequencing efficiencies, and bioinformatic filtering (Deagle et al. 2013; Lamb et al. 2016; McLaren et al. 2019; Thomas et al. 2016). Ideally, the relationship between a species' actual proportion in mass (PROP_mass_) and its proportion of reads (PROP_reads_) in the sequencing output would have a slope of one, so each one‐unit increase in the proportion of mass results in a one‐unit increase in the proportion of reads. However, this is rarely the case because of bias introduced in the processing pipeline. Fortunately, when using internal standards (the same quantity of a control species added to each sample), calibration mixtures (mock mixtures with known species composition and species relative abundances), researchers can make better predictions of a species' relative abundance and correct for introduced bias when characterizing ecological assemblages (Harrison et al. 2021; Matesanz et al. 2019; McLaren et al. 2019; Thomas et al. 2016). Other studies have used calibration mixtures to calculate a correction factor, a number that is derived from the actual PROP_mass_ of species in the calibration mixture and the PROP_reads_ detected. This correction factor can improve mixture composition estimates when certain species in the mixture are disproportionately represented in sequencing output (Matesanz et al. 2019; Thomas et al. 2016; Zhu et al. 2023).

Often in plant ecology, studies occur across multiple years to evaluate how environmental variation between years influences plant community composition or the ecosystem functions they provide. Additionally, when working with roots, a common question is how plant community composition varies across depths in the soil. However, one key knowledge gap limiting our ability to quantify root biomass of distinct species accurately is whether and to what extent the association between PROP_reads_ and PROP_mass_ varies by year and depth, as well as how the presence of non‐target species (i.e., weeds or root material from previously growing plants) influences this association. Plant roots are plastic, responding to the environmental conditions, soil characteristics and plant community in which they grow (Amsili and Kaye 2020; Bardgett et al. 2014; Freschet et al. 2021; Rucińska et al. 2021; Xia et al. 2022). This influences their biomass production, but also the lignin content and other chemical components of root tissues (Fisk et al. 2010; Rahman et al. 2020; Shamloo et al. 2017; Sharma et al. 2020), which can influence both DNA extraction and PCR amplification efficiencies in mixed plant samples. This potentially results in differing amounts of bias across years that vary in environmental conditions. Additionally, mixtures used to calibrate the relationship between PROP_mass_ and PROP_reads_ are created from species monocultures, but plant communities often contain a certain proportion of weeds and DNA from root tissue of previously grown plants. These factors can contribute to bias and influence the association between a species' PROP_mass_ and PROP_read_ in the final sequencing output; however, it is not yet clear whether including these explanatory variables in models can help improve predictions of a species proportions within total root mass.

Accurately modelling a species' PROP_mass_ based on its PROP_reads_ in the sequencing output from calibration mixtures is crucial for predicting the abundance of diverse plant species in belowground biomass (Matesanz et al. 2019; Thomas et al. 2016). However, this calibration process is both costly and time‐consuming, so there is a need to determine the minimal effort needed to model the relationship between species PROP_reads_ and actual species PROP_mass_. Currently, the optimal number of calibration points required to accurately predict a species' PROP_mass_ is unknown, and researchers often do not include a rationale behind the chosen number of calibration samples (Matesanz et al. 2019; Thomas et al. 2016; Wagemaker et al. 2020; Zhu et al. 2023). Additionally, it is not known how many reads per sample are needed for accurate species predictions. By determining the optimal requirements for the number of calibration samples and reads per sample, we can enhance this method's application in high‐throughput projects.

In this study, we modified a metabarcoding method to determine the relative composition of plant species within the root biomass of diverse mixtures. To build and improve upon the work of others, our first objective was to evaluate whether including explanatory variables (year, depth, treatment, and non‐target proportion of reads (NTPR)) in our models improved prediction accuracy. Because the relationship between a species' PROP_mass_ and PROP_reads_ is essential to obtaining accurate predictions of a species' root biomass in mixtures, our second, complementary objective was to determine to what extent the PROP_mass_—PROP_reads_ relationship varies by year, depth and NTPR. Our third objective was to determine the optimal reads per sample and calibration mixture number required for accurate PROP_mass_ estimates, while minimizing resource investment. Understanding how the PROP_mass_—PROP_reads_ relationship varies across sources of environmental variation in ecological studies, such as those that occur across different years and soil depths, is essential for quantifying the composition of distinct plant species in root biomass.

## 2 Materials and Methods

### 2.1 Experimental Design and Site Description

To address our research goal of predicting a species' proportion in root mass from its proportion in reads, we used four cover crop monocultures from a long‐term cover crop diversity experiment at the Russell E. Larson Agricultural Research Center in Rock Springs, PA, USA (40°43′ N, 77°55′ W) in the Fall of 2021, 2022, and 2023. Site temperature and precipitation data are summarized in Table S1 (Soil Survey Staff, Natural Resources Conservation Service 2025a). Soils are primarily comprised of Murrill channery silt loam (fine‐loamy, mixed, semiactive, mesic Typic Hapludult), with Hagerstown silt loam and Buchanan channery loam on the remaining 15% of the site (Soil Survey Staff, Natural Resources Conservation Service 2025b).

Cover crops are planted between cash crops to provide ground cover, ecosystem services, and soil health benefits. Each year, the field was mowed and chisel‐ploughed before cover crop planting. Plant seeding rates are described in Table S2. Dates of field operations and sample collections are in Table S3. The cover crop mixture experiment uses a randomized complete block design with four replications of diverse cover crop treatments (Kaye et al. 2019; Murrell et al. 2017). Within the larger cover cropping trial, we selected four cover crop monocultures that span three plant functional groups (brassica, legume, grass) of interest. The four cover crop species included in the treatments are canola ( *Brassica napus* L.), crimson clover ( *Trifolium incarnatum* L.), Austrian winter pea ( *Pisum sativum* L.), and triticale (x *Triticosecale* ‘Wittmack’). Cultivar details are in Table S2. After planting each year, we established a one m^2^ subplot for collecting root cores within **t**he cover crop monoculture plots. To reduce the presence of non‐target species, subplots were hand‐weeded two times in 2021; however, to reduce labor requirements, this was reduced to one time per subplot in 2022 and 2023.

### 2.2 Field Sampling and Processing

Root samples were collected in November each year. In 2021 and 2022, we extracted four soil cores (40 cm depth and 10.2 cm diameter; Giddings tractor‐mounted hydraulic probe). However, due to malfunctioning equipment, we switched our deep sampling probe in 2023 and took 12 soil cores (40 cm depth and 5.1 cm diameter; AMS 9110 Ag‐Probe) within each treatment subplot (Figure 1). We separated cores into two depths (0–20 and 20–40 cm), then pooled cores by depth, to quantify root composition above and below the plough line. Roots were cleaned using a hydropneumatic elutriation system (0.5 mm sieve, Gillison's Variety Fabrication Inc.), dead roots and non‐root organic matter were removed (i.e., seeds), and roots were then freeze‐dried for 72–96 h (Labconco FreeZone) and ground to powder (GenoGrinder at 1200 RMP for five minutes).

**FIGURE 1:**
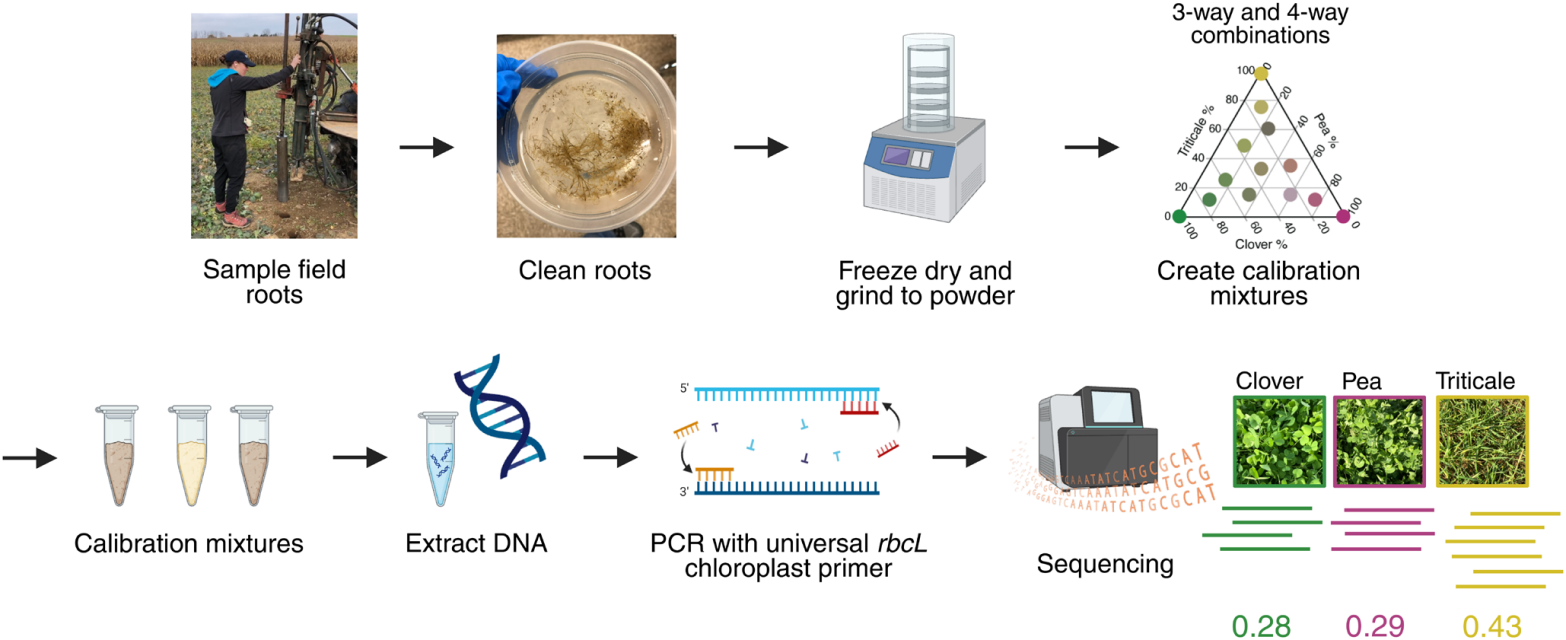
Visual overview of methods used to determine field root mixture composition by species through metabarcoding. Created in BioRender. Rice, E. (2026) https://BioRender.com/u575d42.

For a subsequent study, we wanted to determine the relative abundance of each of our cover crop species in the root biomass of two mixture treatments in the field: a three‐ (crimson clover, Austrian winter pea, and triticale) or four‐species (canola, crimson clover, Austrian winter pea, and triticale) mixture. Therefore, we used the tissue collected from the monoculture plots of our four species of interest to create calibration mixtures with varying levels of known proportions of each species in these three‐ or four‐species combinations. While planted as monocultures, there were non‐target species (e.g., weeds) present in low proportions in each treatment. Each calibration sample included 25 mg of freeze‐dried root tissue with varying proportions of each species in the sample mass. Table 1 summarizes the calibration mixtures across the three years. We created 152 calibration mixtures in 2021 and 108 each in 2022 and 2023. There were between 25 and 60 calibration points per year and depth for all species. After weighing, samples were stored at −40°C. DNA was extracted from all samples using the DNEasy Plant Minikits (Qiagen; catalogue ID: 69106).

**TABLE 1:** Summary of calibration mixtures used to model and verify method accuracy across the three‐year study. Combinations are the number of different species combinations created, and range indicates the percent range of each species mass in mixture within those combinations.

Year	Samples	Mixture treatment	Combinations	Range (%)
2021^a^	80	4 species	9	8–100
	72	3 species	8	10–100
2022^b^	68	4 species	7/8 (depending on block)	4–100
	40	3 species	4	5–100
2023^b^	68	4 species	7/8 (depending on block)	5–100
	40	3 species	4	4–100

^a^ Each species combination was replicated across the two depths and four blocks.

^b^ Each species combination was replicated across the two depths; the composition of species differed across blocks to maximize calibration spread, particularly at higher proportions.

### 2.3 Primer Design

In this study, we aimed to use primers that would lead to substantial overlap between forward and reverse DNA strands when using 250 × 250 base pair sequencing, whereas many common primers in plant DNA barcoding cover larger gene regions (Bell et al. 2017; De Boer 2022; Hollingsworth 2011; Hollingsworth et al. 2016; Kress and Erickson 2007). To test this quantification method across many samples and capture spatial and temporal variation, we focused on a single plant marker. While ITS, *rbcL*, *matK*, and *trnL* were tested in preliminary trials targeting these markers, we found that *rbcL* was the only primer that did not produce multiple bands across all test species. Multiple banding could bias our sequencing reads, leading to the underrepresentation of certain species. Therefore, we proceeded with the *rbcL* chloroplast gene alone, a highly conserved barcoding gene in plants (Bell et al. 2017; Erickson et al. 2008; Hollingsworth et al. 2016; Kress and Erickson 2007; Matesanz et al. 2019).

To describe the process in more detail with *rbcL* specifically, we performed Sanger sequencing of the *rbcL* gene (*rbcL*‐1F: 5′‐ATGTCACCACAAACAGAAAC‐3′, *rbcL*‐724R: 5′‐TCGCATGTACCTGCAGTAGC‐3′); (Fay et al. 1998; Isola et al. 2021) from greenhouse‐grown cover crop cultivars of our four focal species and legacy crops. After several trials with primer design (Geneious Prime version 2022.2) and evaluation for differentiation between species, one primer pair was selected (*rbcL*‐184F: 5′‐GTGGACCGATGGACTTACC‐3′, *rbcL*‐433R: 5′‐AGGAGGACCTTGGAAAGTTTT‐3′). This universal primer only amplifies a single gene region of 250 base pairs and a unique genetic sequence is captured for each of our target plant species.

### 2.4 Amplicon Sequencing

We amplified the *rbcL* chloroplast gene using *rbcL*‐184F and *rbcL*‐433R primers with Illumina overhangs. On 2021 plates, PCR was carried out with a final volume of 25 μL, including 2 μL of template DNA, 0.25 μL of primers (10uM), 12.5 μL of colourless GoTaq Master Mix (Promega; catalogue ID: M7833), 2 μL of 0.08 mg/mL bovine serum albumin (Thermo Fisher Scientific; catalogue ID: B14), and 8 μL of nuclease‐free water. The thermal cycle profile consisted of 95°C for 5 min; 30 cycles of 95°C for 30 s, 52°C annealing temperature for 30 s, 72°C for 40 s; and a final elongation step of 72°C for 7 min. With 2022 and 2023 plates, the PCR recipe changed slightly to contain 1 μL of template DNA, 0.5 μL of primers (10uM), 12.5 μL of Q5 hot start high fidelity 2× master mix (New England Biolabs; catalogue ID: M0494L), 2 μL of 1 mg/mL bovine serum albumin and 8.5 μL of nuclease‐free water. The thermal cycle profile consisted of 98°C for 30 s; 30 cycles of 98°C for 10 s, 52°C annealing temperature for 30 s, 72°C for 30 s; and a final elongation step of 72°C for 2 min. For Fall 2021 plates, PCR product was checked on a gel, magnetic bead cleaned (Omega Bio‐Tek; catalogue ID: M1378‐01), and sent to the Penn State Genomics Core for attachment of index sequences and equimolar sample pooling. For 2022 and 2023 plates, no primer‐dimers or other contaminants were detected on the gel; therefore, bead cleaning was not performed before submitting plates to the Penn State Genomics Core (RRID:SCR_023645). Negative controls were sequenced with 2022 and 2023 Illumina runs.

DNA libraries were constructed using IDT for Illumina DNA/RNA Unique Dual Indexes. Library concentration was normalized using SequalPrep Normalization Plates (ThermoFisher; catalogue ID: A1051001) with sequential elution. Library pool concentration was determined by qPCR using a Kapa Library Quantification Kit (Roche). The library pool was sequenced using a NextSeq 2000 P1 600 cycle kit and performed 300 × 300 paired‐end sequencing using 40% PhiX.

### 2.5 Sequencing Analysis

Computational analyses were performed on the Penn State supercomputer interface, ROAR Collab, with the DADA2 pipeline (version 1.30.0; Callahan et al. 2016) in R (R Core Team 2017) to trim, denoise, and remove chimeric sequences before amplicon sequence variant (ASV) identification and assignment to species. In the second round of sequencing, we did not bead clean and had more non‐specific priming than in the first sequencing run despite gels appearing clean. To address this, ASVs were removed when the sequence length was above or below 250 base pairs. We assessed whether selecting ASVs at 210 base pairs altered the proportions by treatment for each species and found no meaningful difference. Taxonomy was assigned with exact matching (i.e., 100% identity) using a constructed reference database comprised of Sanger sequencing and GenBank *rbcL* gene region sequences (Table S4; Data S1). *RbcL* reference libraries are available online, but they are not yet fully comprehensive (i.e., regional, habitat, or plant group specific); we therefore needed to create our own local reference database of plants commonly found at our site. In total, there were 26 species, including our four cover crops, four legacy crops, and 18 common weeds found in central Pennsylvania. Metadata, ASV, and taxonomy files were combined with *phyloseq* (McMurdie and Holmes 2013), and the output was sorted to determine the proportion of reads for each species per sample. Samples with unknown genus and species identifications were retained and grouped within the weed category. Unknown ASVs accounted for very little of the total proportion of reads in both sequencing runs (0.0004 in the 2021 and 0.004 in the joint 2022 and 2023 sequencing run). Weed and legacy crop categories were combined and called non‐target species.

### 2.6 Statistical Analysis

Our first question was to what extent we can predict PROP_mass_ from PROP_reads_. In other words, how accurately can we predict species root mass from field mixtures using sequencing data? We used a generalized linear mixed model with a beta distribution and logit transformation (glmmTMB package in R, v1.1.11; Brooks et al. 2017), which is commonly recommended for proportional data, since values are constrained between zero and one (Douma and Weedon 2019). Potential explanatory variables in the models included the target cover crop species PROP_reads_, year (2021, 2022, 2023), depth (0–20, 20–40 cm), treatment (three‐ or four‐species mixture), and non‐target PROP_reads_ (NTPR). Block was always included as a random variable. To select the best model for each species with the above variables, we used five randomly divided training (75% of the dataset) and test (25% of the dataset) set evaluations. Each training set was fit to the global model (Table S5), and the dredge function from the *MuMin* package was used to select the model with the lowest AICc value (v1.48.11; Bartoń 2025). The top three models within two AICc units were used with the training and test sets to predict a species PROP_mass_ in a mixture. We then calculated the root mean squared error (RMSE), mean average error (MAE), and conditional *R* ^2^, and the model that consistently produced the highest prediction accuracy across the five training and test iterations was chosen as the final model and fit with the full dataset (hereafter referred to as final model).

Following model prediction with the full dataset and the final models, we fit a linear model between the actual species mixture PROP_mass_ and the predicted species PROP_mass_. If the prediction is 100% accurate, the slope would be near one with an intercept near zero. We also evaluated base models (models with only PROP_reads_ as the explanatory variable) to see how well our predictions improved with the explanatory variables in the final models.

Additionally, we wanted to understand how the relationship between PROP_reads_ and PROP_mass_ varied across years, depths, and non‐target species, without the other variables complicating the model. To do this, we constructed a series of simple models with year, depth, and NTPR individually and the interaction with each explanatory variable and species PROP_reads_. (e.g., species PROP_mass_ ~ species PROP_reads_ * year + (1|block)). We again used generalized mixed effect models with a beta distribution and logit transformation.

Simulations to determine the optimal number of reads and calibration points for accurate predictions:

To examine how prediction accuracy is influenced by sampling, we manipulated the number of total reads and the number of calibration levels used per species, which comprise the greatest expenses in executing this method, including sequencing costs and the exorbitant amount of time required to create calibration mixtures.

To determine the minimum number of reads per sample (RPS) required to obtain accurate species root mass predictions, we ran repeated simulations with a random selection of reads from each sample, starting at 35,000 RPS and reducing to 100 RPS (i.e., *in silico* downsampling of the sequencing data). We systematically reduced the read number by 5000 until reaching 10,000 RPS, then by 1000 until reaching 1000 RPS, and finally by 100 until reaching 100 RPS. Each simulation involved processing the downsampled sequencing data with the same bioinformatics pipeline as we used for the entire sequencing dataset, splitting the samples into training (75% of samples) and test (25% of samples) datasets, recalculating parameter estimates (i.e., betas and intercept) for the best predictive model for each species previously determined with the training dataset, and calculating prediction accuracy (*R* ^2^ and RMSE) with the test dataset. Each iteration of read number simulations was repeated 500 times to generate a distribution of possible *R* ^2^ and RMSE values. To test if this method was better than random, within each simulation, we also created a ‘randomized’ dataset with the proportion of reads data shuffled between samples, resulting in no relationship between species PROP_mass_ and species PROP_reads_, and calculated *R* ^2^ and RMSE, resulting in expected null distributions for these statistics.

To determine the optimal number of calibration mixture samples needed for each species, we simulated stepwise increases in the number of calibration points within set ranges of PROP_mass_, ranging from a minimum of two to a maximum of 11 proportions of an individual species in root mass used for calibration (Table S6). For example, for a two‐level simulation, we included two points randomly selected within set ranges (0–0.1 and 0.6–1 as the proportion of an individual species in root mass), which meant we had 12 total calibration samples for that species (two levels * three years * two depths). Calibration samples were used as the training dataset for model fitting (again using the previously determined best predictive model for each species), and then the full dataset was used as the test dataset to compare predicted PROP_mass_ to actual PROP_mass_ to generate *R* ^2^ and RMSE. This process was repeated 500 times to generate *R* ^2^ and RMSE distributions. To test if this method was better than random, within each simulation, we also created a ‘randomized’ dataset with the proportion of reads data shuffled between samples, resulting in no relationship between species PROP_mass_ and species PROP_reads_, and calculated *R* ^2^ and RMSE, resulting in expected null distributions for these statistics. We subsampled 10,000 reads per species and used the same bioinformatics pipeline as we used for the entire sequencing dataset before the calibration number simulations were run.

## 3 Results

### 3.1 How Accurately Did Sequence Proportions Predict Actual Relative Abundance in Root Biomass?

While the best predictive model varied across the four species (Table 2), our ability to predict a species' relative mass (PROP_mass_) based on the PROP_reads_ was fairly accurate (*R* ^2^ values between predicted and actual species mass range from 0.73 to 0.86; Figure S1, Figure 2), however, the accuracy of these predictions depended on multiple factors including year, depth, and non‐target PROP_reads_ (NTPR). Interestingly, we found that treatment (different species mixture combinations) did not account for any variation in our models across species; therefore, this variable was removed during the final model selection process.

**TABLE 2:** The best explanatory variables and variable interactions selected for each species to predict its proportion in mass and their associated root mean square error (RMSE), mean average error (MAE), and *R* ^2^ fit statistics.

Species	Final model	RMSE	MAE^a^	*R* ^2^
Canola	canola.prop.reads + year + depth + non.target.prop.reads + year:canola.prop.reads + depth:canola.prop.reads + canola.prop.reads:non.target.prop.reads + year:depth:canola.prop.reads	0.12	0.08	0.86
Clover	clover.prop.reads + year + depth + non.target.prop.reads + clover.prop.reads:non.target.prop.reads	0.16	0.11	0.73
Pea	pea.prop.reads + year + depth + non.target.prop.reads + year:pea.prop.reads + pea.prop.reads:non.target.prop.reads	0.13	0.10	0.79
Triticale	triticale.prop.reads + year + non.target.prop.reads + year:triticale.prop.reads + triticale.prop.reads:non.target.prop.reads	0.14	0.11	0.77

*Note:* Block was included as a random effect in all models.

^a^ RMSE is a more common measure of prediction error; however, MAE is an unweighted measure that reduces the influence of outliers.

**FIGURE 2:**
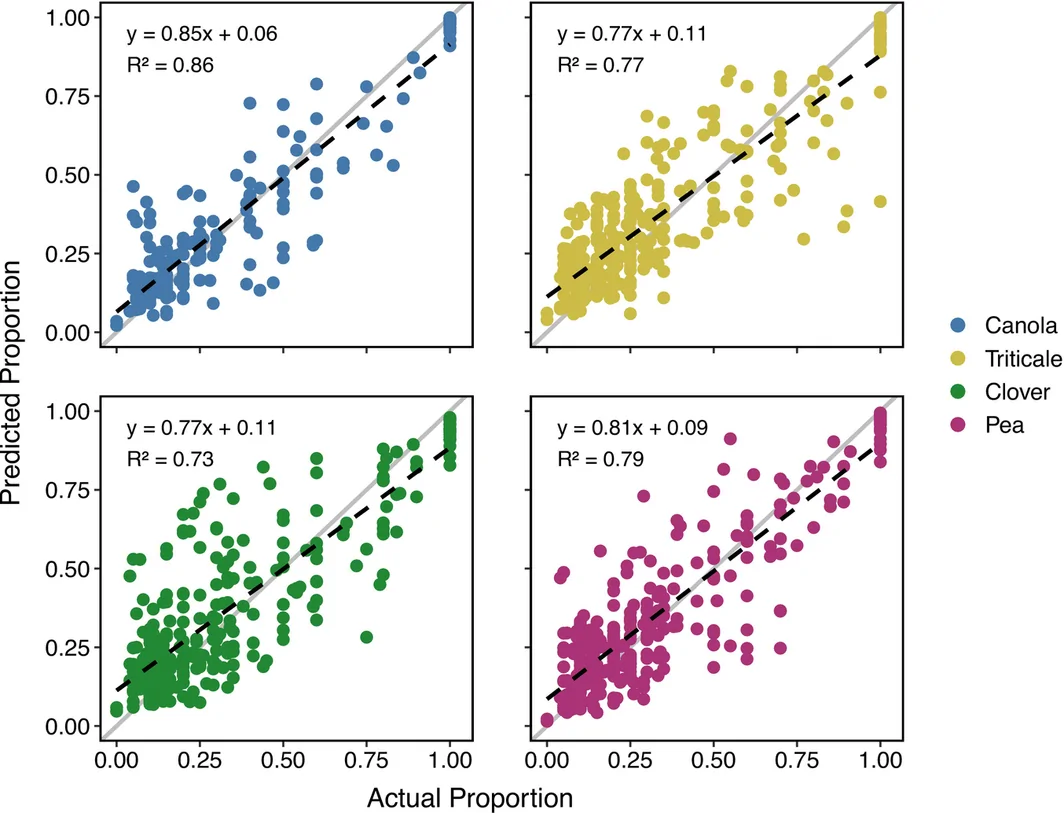
Relationship between actual species mass proportion in the calibration mixture and predicted species mass proportion in mixture based off models in Table 2. The point colour corresponds with species, solid grey line is the target 1:1 relationship with a slope of 1 and intercept of 0, the dashed black line is the fitted linear model. The model *R* ^2^ and linear model equation are shown for each species.

The variation in prediction accuracy across species was minimal; canola had the smallest RMSE and MAE, closely followed by pea, triticale and clover (Table 2), with all RMSE values falling within 0.04 of one another and MAE values within 0.03. The difference between the predicted and actual PROP_mass_ varied by species and ranged from 0 to 0.41 (average difference of 0.08) for canola, 0 to 0.48 (average difference of 0.11) for clover, 0 to 0.45 (average difference of 0.1) for pea and from 0 to 0.58 (average difference of 0.11) for triticale. Across our four target species, the slope ranged from 0.77 to 0.85 and the intercept ranged from 0.06 to 0.11. When comparing the RMSE, MAE, and *R* ^2^ between the final models and the base models for each species, which only included PROP_reads_ as a predictor variable, we found, as we expected, that the prediction accuracy was consistently better with the final models that included additional explanatory variables (Table 2 compared to Table S7). For example, when including the explanatory variables of year, depth and NTPR, the *R* ^2^ between actual PROP_mass_ and predicted PROP_mass_ increased from 0.68 to 0.86 for canola, 0.6 to 0.73 for clover, 0.37 to 0.79 for pea and 0.36 to 0.77 for triticale.

Year and NTPR were the two explanatory variables that explained the most variability and were consistently included in the final models. Year, NTPR, and the interaction of each explanatory variable with PROP_reads_ were included in the final models for canola, pea, and triticale. The one exception was clover, where the year had an additive effect only, but NTPR and its interaction with PROP_reads_ were still included (Table 2). Depth explained less variability in our mixture samples across species. From our training and test set evaluations, both depth and the interaction of depth with PROP_reads_ were only included in canola's final model. Clover and pea's final models included depth as an additive effect but lacked the interactions between depth and PROP_reads_, while triticale did not include depth at all (Table 2).

### 3.2 To What Extent Did the Proportion in Reads and Mass Vary Across Years, Depth and Non‐Target Proportion of Reads?

Year, NTPR, and to a lesser extent, depth, were important predictors of the PROP_mass_ across all species; we examined to what extent the relationship between PROP_reads_ and the actual PROP_mass_ varied across each of these explanatory variables individually. Additionally, because creating calibration mixtures for each sampling depth and year is time‐intensive and costly, we examined how accurate our predictions would be if we used one level (e.g., one year or one depth) to calibrate and predict across all years and depths.

#### 3.2.1 Year

The relationship between PROP_reads_ and PROP_mass_ varied significantly between years for all four cover crop species (Table 3, Figure 3). For certain years, we saw large differences. For example, when the actual PROP_mass_ of a species increased from 0.4 to 0.6 in the calibration mixtures, the corresponding increase in PROP_reads_ in pea was 0.03 in 2021 and 0.07 in 2023, more than a two‐fold increase. Likewise, for triticale, an increase in actual PROP_mass_ from 0.4 to 0.6 resulted in a 0.07 increase in PROP_reads_ in 2021, but a 0.01 increase in 2023, a seven‐fold difference between the two years of the study.

**TABLE 3:** Results from generalized linear mixed models evaluating whether the association between proportion of reads and proportion of mass varied across years for our four species of interest.

Year effects								
	Canola		Clover		Pea		Triticale	
	Chisq	*p*	Chisq	*p*	Chisq	*p*	Chisq	*p*
Year	4.37	0.11	5.30	0.07	61.39	**< 0.001**	60.83	**< 0.001**
Prop.reads	127.37	**< 0.001**	243.19	**< 0.001**	90.93	**< 0.001**	280.49	**< 0.001**
Prop.reads:Year	23.15	**< 0.001**	18.05	**< 0.001**	0.59	0.74	60.21	**< 0.001**

*Note:* Model: species.mix.prop ~ species.prop.reads * year + (1|block). Statistically significant *p* < 0.05 are shown in bold.

**FIGURE 3:**
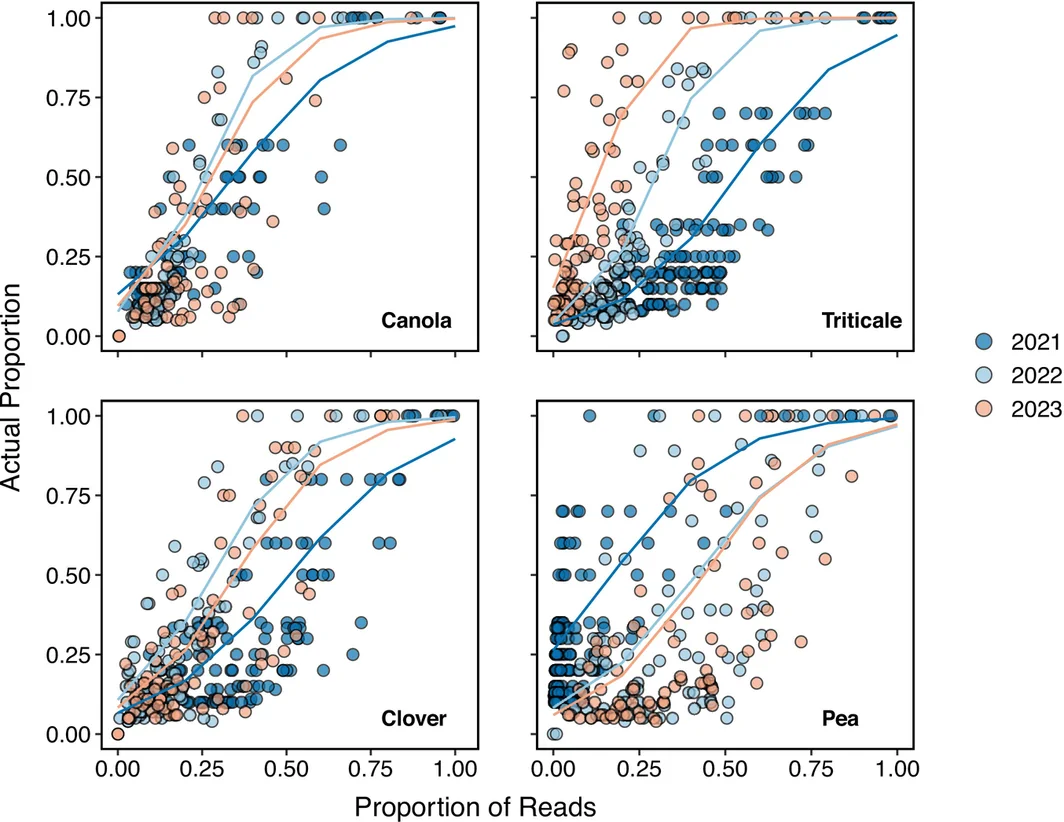
The relationship between species proportion of reads and actual species mass proportion in the calibration mixture across three sampling years with the simple model. Statistical results in Table 3. The point and trend line colour correspond with year and each panel is a different species in the mixture.

When we calibrated the model from a single year and predicted PROP_mass_ for all three years, we found that in general, the predicted proportions were widely dispersed, resulting in many highly inaccurate predictions of species mass in mixtures across all three years of the study (Figures S2–S5). For example, pea's *R* ^2^ values ranged from 0.24 to 0.58 when calibrating by a single year compared to an R^2^ of 0.79 when calibrating with mixtures from all study years (Figure S4). Aligning with the simple model findings, canola and clover were not as affected by year differences and had better predictions with single‐year calibration than the other focal species (Figures S2 and S3).

#### 3.2.2 Depth

The slope between PROP_reads_ and PROP_mass_ varied across depths for canola only, while the additive effect of depth was significant for pea and clover (Table 4, Figure 4). For canola, when the actual PROP_mass_ of a species increased from 0.4 to 0.6 in the calibration mixtures, the corresponding increase in PROP_reads_ was 0.05 at the 0–20 cm depth and 0.03 at the 20–40 cm depth. Likewise, when we calibrated the model with mixtures from a single depth and predicted PROP_mass_ for both depth segments, we found that all *R* ^2^ values were smaller than when both depths were used, but not substantially so (Figures S6–S9). As an example, canola's predicted PROP_mass_ and actual PROP_mass_ *R* ^2^ was 0.83 when calibrated by 0–20 cm depth, 0.7 when calibrated by 20–40 cm depth, and 0.86 when calibrated by both depths. Additionally, the depth that produced the higher *R* ^2^ was not consistent across species; canola and clover had better predictions when calibrated by 0–20 cm depth, while pea was the opposite, with 20–40 cm depth rendering a larger *R* ^2^, and triticale produced the same *R* ^2^ value when calibrated by either depth.

**TABLE 4:** Results from generalized linear mixed models evaluating whether the association between proportion of reads and proportion of mass varied across soil depths for our four species of interest.

Depth effects								
	Canola		Clover		Pea		Triticale	
	Chisq	*p*	Chisq	*p*	Chisq	*p*	Chisq	*p*
Depth	0.69	0.41	4.56	**0.03**	4.32	**0.04**	0.05	0.83
Prop.reads	143.27	**< 0.01**	224.39	**< 0.01**	118.77	**< 0.01**	99.09	**< 0.001**
Prop.reads:Depth	3.51	0.06	2.18	0.14	0.33	0.57	0.03	0.87

*Note:* Model: species.mix.prop ~ species.prop.reads * depth + (1|block). Statistically significant *p* < 0.05 are shown in bold.

**FIGURE 4:**
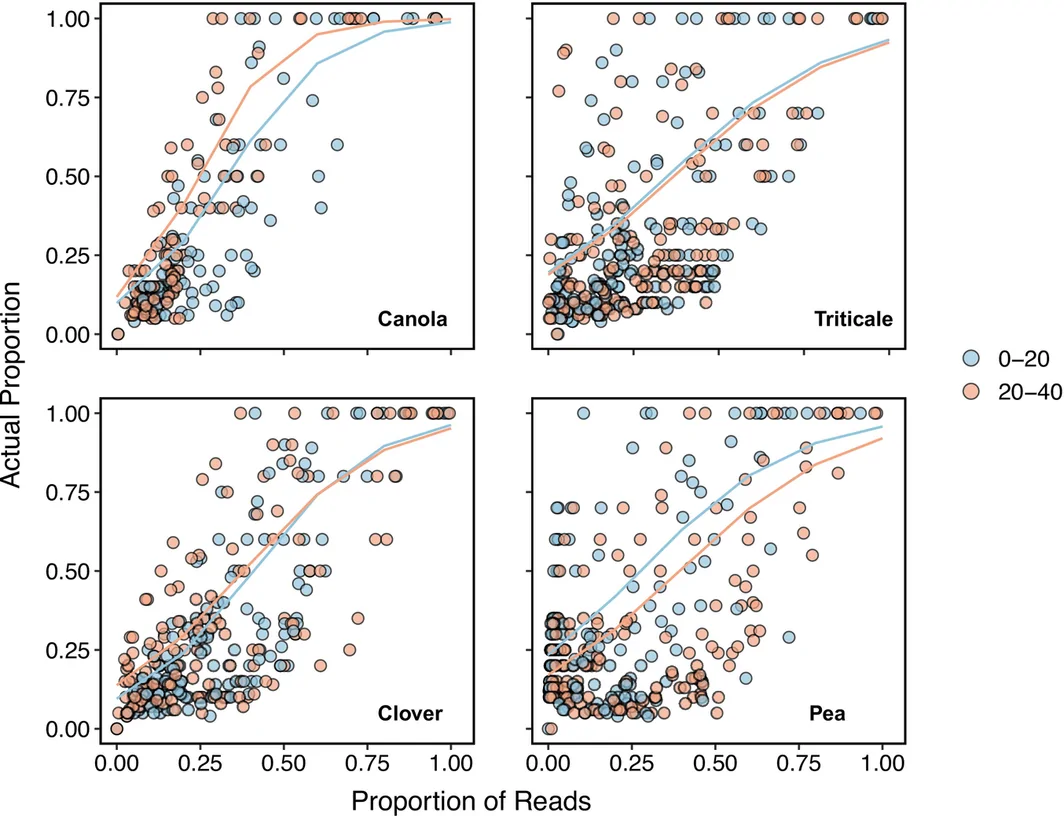
The relationship between species proportion of reads and species mass proportion in the calibration mixture across two sampling depths. Statistical results in Table 4. The point and trend line colour correspond with depth and each panel is a different species in the mixture.

#### 3.2.3 Non‐Target Proportion of Reads

We found that the relationship between PROP_reads_ and PROP_mass_ varied significantly across NTPR (which included both weeds and legacy crops) for each species (Table 5, Figure 5). For example, when the actual PROP_mass_ of a species increased from 0.2 to 0.4 in the calibration mixtures, the corresponding increase in PROP_reads_ in clover was 0.05 in the low NTPR category and 0.1 in the high category, a two‐fold increase. Likewise, for pea, an increase in actual PROP_mass_ from 0.2 to 0.4 resulted in a 0.03 increase in PROP_reads_ at low NTPR and a 0.07 increase at high NTPR, another two‐fold difference between the low and high NTPR ranges.

**TABLE 5:** Results from generalized linear mixed models evaluating whether the association between proportion of reads and proportion of mass varied across non‐target species proportion of reads (NTPR; weeds and legacy crops).

Non‐target species effects								
	Canola		Clover		Pea		Triticale	
	*z*‐value^a^	*p*	*z*‐value	*p*	*z*‐value	*p*	*z*‐value	*p*
NTPR	−3.41	**0.001**	0.61	0.54	0.91	0.36	3.36	**0.001**
Prop.reads	9.63	**< 0.001**	15.12	**< 0.001**	7.75	**< 0.001**	11.38	**< 0.001**
Prop.reads:NTPR	8.56	**< 0.001**	4.95	**< 0.001**	4.85	**< 0.001**	4.25	**< 0.001**

*Note:* Model: species.mix.prop ~ species.prop.reads * non.target.prop.reads + (1|block). Statistically significant *p* < 0.05 are shown in bold.

^a^ Non‐target proportion of reads is a continuous variable resulting in a z‐value statistic.

**FIGURE 5:**
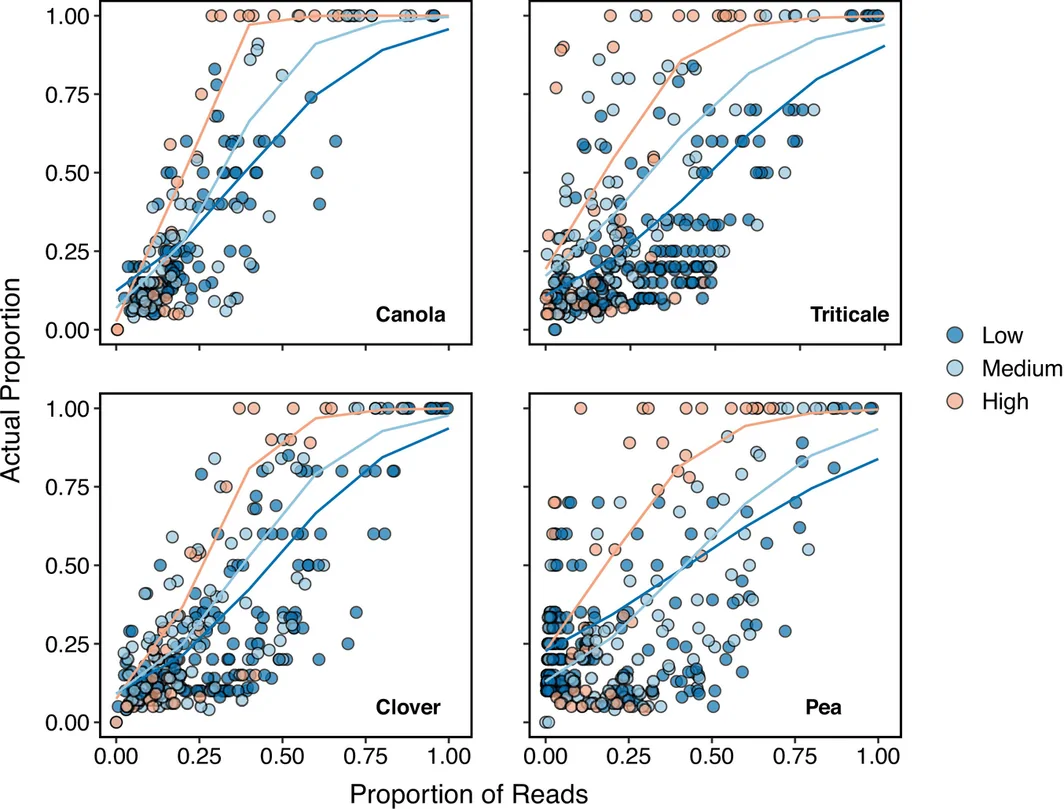
The relationship between species proportion of reads and species mass proportion in the calibration mixture across three non‐target species categories (low 0.0–0.1, medium 0.1–0.3, and high 0.3–1). These categories were constructed for visual purposes only; the statistical results in Table 5 are based on the continuous data. The point and trend line colour correspond with non‐target species categories, and each panel is a different species in the mixture.

The proportion of reads belonging to non‐target species ranged from 0 to 0.88. Weeds dominated the NTPR, yet in 55% of samples the weed proportion was below 0.25. Legacy crops made up less than 0.05 proportion of the relative abundance in 99% of samples. The largest weed proportions were typically found in 2022 and 2023, which can be seen in Figure 3 where the spread of PROP_reads_ of the monoculture samples (when actual proportion is one) is much higher than in 2021, the year in which we hand‐weeded our sampling plots twice instead of once.

### 3.3 How Many Reads Per Sample and Calibration Points Are Required for Accurate Predictions of Species in Simple Mixtures?

We ran a simulation to determine how many reads per sample (RPS) are required for accurate species predictions in mixtures (Figure 6a,c). The highest *R* ^2^ value and lowest RMSE based on RPS differed slightly for each species, but on average, the *R* ^2^ peaked at 700 RPS (*R* ^2^ canola = 0.84, clover = 0.75, pea = 0.77, triticale = 0.75; RMSE canola = 0.13, clover = 0.15, pea = 0.14, triticale = 0.15). However, the difference between *R* ^2^ and RMSE from 700 and 100 RPS was on average only 0.03 and 0.01, respectively, across our four species. Very few RPS are needed for accurate predictions in simple plant mixtures.

**FIGURE 6:**
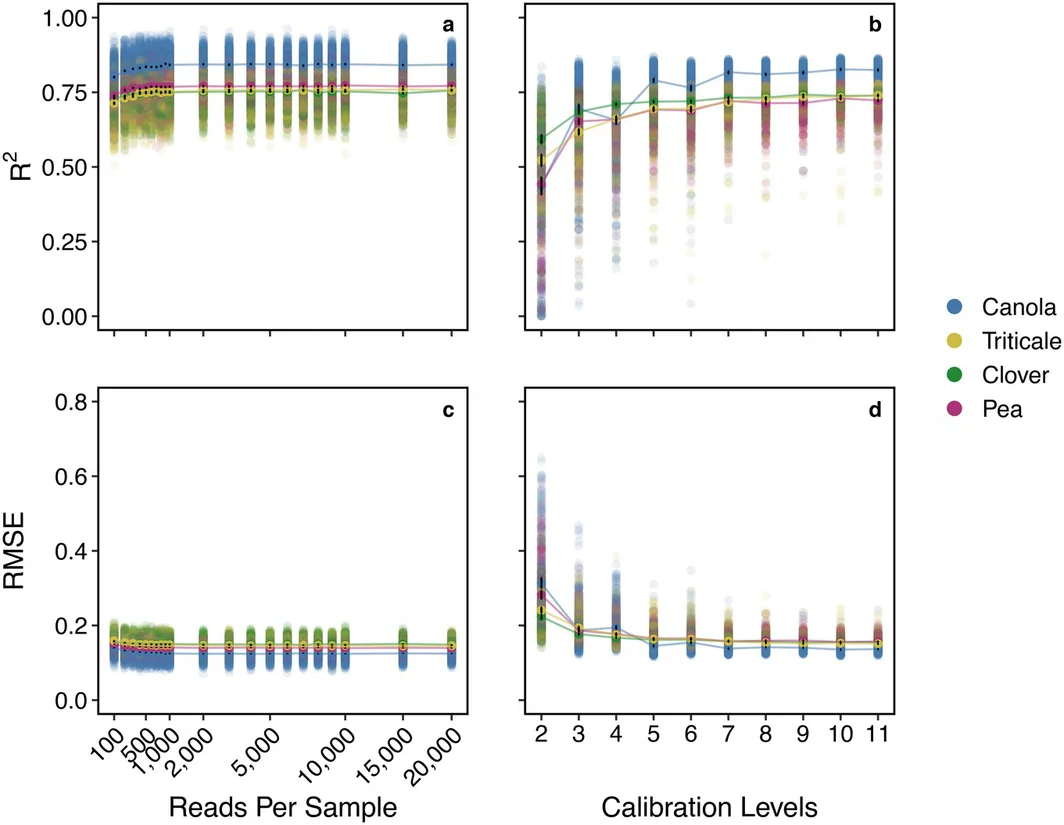
Simulation output with 500 replicates to determine (a) how many reads per sample are needed to attain a high *R* ^2^ and (c) low root mean squared error (RMSE) and 10,000 replicates to determine (b) how many calibration levels are needed for a high *R* ^2^ and (d) low root mean squared error. The point and line colour corresponds with the species. Simulated reads per sample experiment went up to 35,000, but we are only showing up to 20,000 reads here.

To validate these findings, we calculated expected null distributions for both *R* ^2^ and RMSE. As expected, we found a much smaller *R* ^2^ and larger model RMSE measure when the relationship between species PROP_mass_ and species PROP_reads_ was randomized (Figure S10a,c). All species were around 0.14 RMSE with the original dataset, but when randomly generated reads were used, the average RMSE across species was 0.42.

We ran a second simulation to determine the number of calibration points required for accurate species predictions in mixtures (Figure 6b,d). We found a large increase in prediction accuracy from two to three levels per year and depth (*R* ^2^ = 0.5 to 0.66 and RMSE = 0.26 to 0.19, respectively), and above three levels we saw marginal improvements until reaching seven levels when the prediction accuracy plateaued and became more consistent (e.g., there is less spread around the mean; *R* ^2^ = 0.75 and RMSE = 0.15). Given our results that depth was not integral for this study, we could likely use fewer than 42 calibration points (seven levels * three years * two depths) and still achieve accurate predictions.

We calculated expected null distributions as well and found a much smaller *R* ^2^ and larger model RMSE output when the relationship between species PROP_mass_ and species PROP_reads_ was randomized (Figure S10b,d). The average RMSE for all species at seven levels was 0.15 with the original dataset, but when randomly generated reads were used, the average RMSE at seven levels was 0.42.

## 4 Discussion

We aimed to enhance the accuracy of predicting the proportion of individual plant species in root biomass (PROP_mass_) through sequencing‐based approaches (PROP_reads_) by accounting for additional variables in our prediction models. This includes variables that can be determined from sequences alone (i.e., non‐target proportion of reads; NTPR) and those that require re‐calibration across study conditions (e.g., year, depth). In doing so, we also characterized how much the PROP_mass_—PROP_reads_ relationship varies by year, depth, and NTPR. While the final models predicting PROP_mass_ varied by species in our study, incorporating the fixed effects of year, depth, and NTPR generally improved prediction accuracy. A secondary objective was to evaluate the number of calibration samples and sequencing depth necessary to obtain accurate predictions, as high‐throughput application of this approach would benefit from a protocol that is time‐ and cost‐effective. We found that seven calibration levels (or proportional segments) per year and depth (42 total calibration mixture samples), and 700 reads per sample obtained high‐accuracy predictions for simple mixtures.

### 4.1 Calibrating Across Years, NTPR, and to a Lesser Extent Depth, Improves Prediction Accuracy

We found that the PROP_mass_—PROP_reads_ relationship varied substantially between years, depth, and the amount of NTPR for each species of interest, due to variation in the amount and source of bias introduced throughout the study pipeline. This bias largely results from within‐ and between‐species variation in root tissue chemistry, which varies within a species when years or soil depths are characterized by contrasting environmental conditions. McLaren et al. (2019) thoroughly summarize sources of bias, which include differences between species in the quantity of a gene per unit of tissue weight, tissue lysing efficiencies, and preference during DNA extraction, PCR amplification, sequencing and bioinformatic filtering (other sources on this topic: Qin et al. 2023; Silverman et al. 2021; Yeh et al. 2021). This ultimately causes uncontrollable variation in the relationship between PROP_mass_ and PROP_reads_.

In this study, we used the *rbcL* chloroplast gene, which is found in all segments of plants, including roots, where plastids perform a myriad of functions, such as providing tissue structure and storage. For example, amyloplasts store starch, and proteinoplasts store protein in roots (Aronsson and Solymosi 2024; Choi et al. 2021; Sierra et al. 2023). The number of plastids and copies of plastid genes per cell differ between tissue types (e.g., leaves versus roots) and with developmental stage (Greiner et al. 2020), and the plastid number likely differs between tissues of various root types, such as between a taproot and a lateral root. Fisk et al. (2010) found that tree root diameter impacted PROP_read_ output and attributed this to differences in the *trn*L intron plastid gene copy number. Therefore, variation in the number of plastids or copies of plastid genes per milligram of root tissue may have been one source of bias contributing to the variation between PROP_reads_ and PROP_mass_ across years and depths in our study system.

Environmental variation between years can cause variation in plant tissue chemistry, which can influence the efficiency with which root tissues are lysed, DNA is extracted from plant tissue, and PCR efficiency. For example, lignin content differs between species (Lourenço and Pereira 2018), and precipitation alters lignin content in plants (Choi et al. 2023; Nguyen et al. 2016). In Fall 2022, the site experienced lower precipitation than in the other years of our study, which may have increased lignin content at differing rates across our target species. Increased lignin content in plant tissue can decrease DNA extraction efficiency, potentially resulting in less DNA extracted from one species relative to the root tissue of other species in the mixture. Likewise, lignin and other recalcitrant plant tissues can contain PCR‐inhibiting compounds, potentially decreasing PCR efficiency with increasing proportions of a species with greater lignin content. While we cannot pinpoint the exact source of bias within our study system, such as lignin content or plastid number in the root tissue of our target species, future research may improve the PROP_mass_ prediction accuracy by quantifying potential biochemical sources of bias.

Variation in management and sampling could have influenced year‐to‐year differences. For example, the reduction in weeding frequency in 2022 and 2023 may have affected the physiology of target species by altering interspecific competition. Such effects could influence root biochemical composition (e.g., lignin content), potentially contributing to differences across years. Additionally, due to equipment failure in Fall 2023, smaller‐diameter cores were collected and may have influenced the efficiency of root recovery. While the number of cores was increased to compensate for the core diameter change, the composition of root types (e.g., taproot versus lateral root) may have changed, influencing lignin content and plastid number, adding to the variation across years.

While depth was included in all final models aside from triticale, it explained less variance than other predictor variables. Interestingly, depth was included in the models for canola, pea, and clover, all taprooted species, but not for triticale, which has a fibrous root system. Out of the taprooted species, canola's taproot is larger in diameter and deeper rooting (Gan et al. 2009; Amsili and Kaye 2020). Taprooted species such as canola may be more likely to have variation in root lignin content and chemical composition between root tissue collected at different depths (Prieto et al. 2015, 2016; Suseela et al. 2020), especially as the diameter of the taproot can substantially vary between depths. This may explain why canola was the only species with both depth and the interaction between depth and canola PROP_reads_ in the final model. It is possible that we would have seen greater variability between depths if we had sampled deeper than 40 cm, especially if soil physical properties substantially varied at deeper depths. However, sampling beyond 40 cm was challenging for our study site, as our soil is relatively shallow and extremely rocky.

We also found that incorporating the PROP_reads_ from non‐target species (NTPR), such as weeds and legacy plants, which are an inherent component of field samples, improved our prediction accuracy. Despite our efforts to remove non‐target plant species from our monoculture experimental subplots (i.e., weeding) to reduce mixture complexity, removing all weed and legacy crop contamination from field‐collected root cores is impossible. Unsurprisingly, the largest weed proportions were typically found in 2022 and 2023 compared to 2021, likely due to reduced weeding intensity from twice to once a season in the sampling subplots in these years. However, the degree of weediness can vary across plots; in our study, NTPR ranged from 0 to 0.88 across our four monoculture treatments over the three‐year study. Interestingly, larger weed aboveground biomass did not always coincide with high NTPR, for example the highest aboveground weed proportion (0.12 from a pea plot in 2023) resulted in a proportion of NTPR of 0.26, a relatively medium value (Figure S11). In general, slower‐growing monoculture treatments (e.g., legumes) tend to be weedier than their mixture counterparts due to reduced biomass and competition (Baraibar et al. 2018; Blesh et al. 2019; Connolly et al. 2018). We found no relationship between NTPR and prediction accuracy for any of our species of interest, suggesting that even weedy samples can be accurately predicted and not accounting for nontarget proportion of reads is just as likely to underestimate as overestimate a species PROP_mass_ (Figure S12).

Overall, after including the explanatory variables of year, depth, and NTPR in our models, the predictions produced by our final models were fairly accurate. However, it's important to note that some of our predictions of root sample composition were inaccurate (e.g., in the most extreme case our model predicted that the proportion of triticale was 0.42 when it was actually 0.99). This could be due to bias introduced at any step in the processing pipeline from root collection to bioinformatic analysis. For example, others have shown that using mock communities can be error‐prone due to imprecise mixture weighing (Silverman et al. 2021). In our study, the three‐ versus four‐species mixture (treatment) did not explain a significant amount of variation, indicating that the presence of an additional species did not affect the association between actual PROP_mass_ and PROP_reads_. This is a major concern in metabarcoding studies, as some species can consistently dominate the PROP_reads_ output, resulting in other species' proportions being artificially small (Hassan et al. 2019; McLaren et al. 2019; Silverman et al. 2021). This indicates that, at least for our study, we could use the four‐species calibration mixtures to predict species mass in a three‐species treatment. However, all four of our species were similar in that they were winter annuals planted at the same time. It is possible that for studies with more diverse plant species that vary in age and life history (e.g., woody perennials), the presence of certain species may alter the relationship between PROP_mass_ and PROP_reads_ for other species in the mixture.

### 4.2 Effort and Resource Investment for Accurate Species Relative Abundance Predictions

Since the cost of sequencing and the time investment in creating mock mixtures for calibration are both potential barriers to the frequent and high‐throughput application of this approach, we evaluated how many reads per sample and how many calibration mixtures are needed for accurate species mass predictions from three‐ and four‐species mixtures. We found that 100 reads per sample resulted in nearly equally accurate predictions as when many more reads per sample are produced. For this study, relatively few reads per sample were needed to produce accurate predictions at a fraction of the cost.

Making calibration mixtures is time‐intensive, so it would be helpful for future researchers to know how best to allocate their time and resources. We created 368 calibration mixtures across our three‐year, two‐depth study and used simulations to determine how many calibration mixtures are needed for model fitting and accurate species mass predictions. We found that only three levels per year and depth combination were needed for high *R* ^2^ and low RMSE outcomes (18 calibration points), but we consistently had high prediction accuracy at seven levels (42 calibration points). Other studies have used a wide range of calibration mixture numbers ranging from five to 111 (Bell et al. 2019; Diaz‐Real et al. 2015; Garrido‐Sanz et al. 2022; Hiiesalu et al. 2012; Lamb et al. 2016; Matesanz et al. 2019; Pornon et al. 2016; Rucińska et al. 2022; Thomas et al. 2016; Wagemaker et al. 2020; Zhu et al. 2023). Collecting and processing field roots is a lengthy and demanding process. To our best estimates, we spent a minimum of five minutes collecting, splitting, and bagging each soil core, 30 min washing and hand‐picking roots from each soil core segment, 15 min weighing and grinding each sample, and 20 min precision weighing a calibration mixture sample. Collectively, that is just over an hour of processing per sample to make a single calibration mixture and a total of around 500 h of work for this study. Most of the time input for this method is spent on root processing and calibration mixture creation. In comparison, our best estimate is that it only required 60 additional hours to process samples from DNA extraction through to sequencing. Looking forward, it would be advantageous for other studies to explore the minimum number of calibration samples required for more complex plant communities and different study systems to guide future researchers.

## 5 Conclusion

Our study supports others' findings that amplicon sequencing can be used to determine the relative abundance of plant species in root mixtures (Hassan et al. 2019; Matesanz et al. 2019). We found the combination of species PROP_reads_, year, non‐target proportion of reads, and (to a lesser extent) depth differed in their explanatory power to achieve the best predictions of PROP_mass_ for each species. Additionally, in our simple mixtures we determined that 42 calibration mixtures (seven levels per year and depth) produced high‐accuracy model calibration. However, it may be challenging to achieve this level of calibration with all focal species in a more diverse plant community; fortunately, our research suggests that relatively accurate predictions can be obtained with as few as 18 calibration samples (three levels per year and depth). Researchers will need to make an informed choice of which of their species to focus on for calibration based on preliminary investigation into likely sources of bias in their amplicon pipeline and accept some reduction in prediction accuracy. However, our study demonstrates that even when non‐target proportion of reads are relatively high in “monoculture” samples which are used to make the calibration mixtures, prediction accuracy is still quite robust. Finally, in this study we were able to differentiate between our species of interest with the *rbcL* gene alone, but it is recommended to use a combination of loci to increase species identification in more complex mixtures (Erickson et al. 2008; Bell et al. 2017; CBOL Plant Working Group 2009). This method assessment is intended to support other metabarcoding studies to make informed choices on the number of calibration mixtures to sequence, what sequencing technology to use, and experimental design to implement to effectively model sample variation and optimize prediction accuracy in varying degrees of community complexity.

## Author Contributions

The study was designed by Emma K. Rice, Carolyn J. Lowry, and Liana T. Burghardt. Jason P. Kaye provided support with field methodology and site management. Terrence H. Bell provided support with laboratory methodology and bioinformatic pipelines. Field and laboratory work was conducted by Emma K. Rice. Bioinformatic and statistical work was conducted by Emma K. Rice, Carolyn J. Lowry, and Jason C. Keagy. Carolyn J. Lowry provided supervision throughout the project. The original manuscript draft was written by Emma K. Rice, Carolyn J. Lowry, and Jason C. Keagy. All authors further reviewed and edited the manuscript.

## Funding

This work was supported by the USDA National Institute of Food and Agriculture grants No. 2023‐67011‐40329 and 2023‐67019‐39791, as well as support from USDA NIFA Hatch Appropriations under project No. PEN04759, PEN04768, and PEN04760, and accession No. 1025327, 1026660, and 1025611, respectively. Additional support was provided by the Natural Sciences and Engineering Research Council of Canada through a Discovery Grant (RGPIN‐2023‐03411).

## Disclosure

Benefits‐sharing statement: Benefits from this research accrue from the sharing of our data and results on public databases as described above.

## Ethics Statement

Our work follows all ethical and research integrity standards.

## Conflicts of Interest

The authors declare no conflicts of interest.

## Supporting information


**Table S1:** Rock Springs, PA average temperature (°C) and cumulative precipitation (cm) summarized monthly and for each year's study duration. Data from NRCS National Weather and Climate Center site number 2036.
**Table S2:** Cover crop focal species abbreviations, planted cultivars, and seeding rates. These seeding rates have been used at the site from 2019 onward.
**Table S3:** Dates of field operations and sample processing.
**Table S4:** Reference database metadata. If no accession number is listed, the sequence was derived from a local sample and Sanger sequencing.
**Table S5:** A global model was input into the dredge function from the MuMin package in R, from which the best model for each species was selected based on predictive accuracy.
**Table S6:** Simulation levels in calibration simulation to determine how many levels are needed to predict species proportions in simple mixtures accurately. Within each level, all years, depths, and treatments are represented if possible (there were some proportional ranges where we did not have all variables present to model).
**Table S7:** Base model with only proportion of reads as the explanatory factor and block as a random factor for each species to predict the proportion mass of a species in an unknown mixture. Reported each models associated fit statistics: root mean squared error (RMSE), mean average error (MAE) and *R* ^2^.
**Figure S1:** The relationship between the actual species mass proportion in the calibration mixture and proportion of sequencing reads. The point colour corresponds with species; the solid grey line is the target relationship with a slope of one and an intercept of zero.
**Figure S2:** The relationship between actual canola mass proportion and predicted canola mass proportion of all samples across years in mixture when calibrating by individual years and all years (calibration year labelled on each plot). The solid grey line is the target relationship with a slope of one and intercept of zero; the dashed black line is the fitted linear model.
**Figure S3:** The relationship between actual clover mass proportion and predicted clover mass proportion in mixture when calibrating by individual years and all years (calibration year labelled on each plot). The solid grey line is the target relationship with a slope of one and intercept of zero; the dashed black line is the fitted linear model.
**Figure S4:** The relationship between actual pea mass proportion and predicted pea mass proportion in mixture when calibrating by individual years and all years (calibration year labelled on each plot). The solid grey line is the target relationship with a slope of one and intercept of zero; the dashed black line is the fitted linear model.
**Figure S5:** The relationship between actual triticale mass proportion and predicted triticale mass proportion in mixture when calibrating by individual years and all years (calibration year labelled on each plot). The solid grey line is the target relationship with a slope of one and intercept of zero; the dashed black line is the fitted linear model.
**Figure S6:** Relationship between actual canola mass proportion and predicted canola mass proportion when calibrating by individual depth segments and all depths (calibration depth labelled on each plot). The solid grey line is the target relationship with a slope of one and intercept of zero, the dashed black line is the fit linear model.
**Figure S7:** Relationship between actual clover mass proportion and predicted clover mass proportion when calibrating by individual depth segments and all depths (calibration depth labelled on each plot). The solid grey line is the target relationship with a slope of one and intercept of zero, the dashed black line is the fit linear model.
**Figure S8:** Relationship between actual pea mass proportion and predicted pea mass proportion when calibrating by individual depth segments and all depths (calibration depth labelled on each plot). The solid grey line is the target relationship with a slope of one and intercept of zero, the dashed black line is the fit linear model.
**Figure S9:** Relationship between actual triticale mass proportion and predicted triticale mass proportion when calibrating by individual depth segments and all depths (calibration depth labelled on each plot). The solid grey line is the target relationship with a slope of one and intercept of zero, the dashed black line is the fit linear model.
**Figure S10:** Simulation of random calibration mixtures to verify that model simulations are better than random chance when using a large dataset. Simulation includes 500 replicates to determine (a) how many reads per sample are needed to attain a high *R* ^2^ and (c) low root mean squared error (RMSE) and 10,000 replicates to determine (b) how many calibration levels are needed for a high *R* ^2^ and (d) low RMSE. The point and line colour corresponds with the species. Simulated reads per sample experiment went up to 35,000, but we are only showing up to 20,000 reads here.
**Figure S11:** Comparison of nontarget species proportion of reads (weeds and legacy crops) and the aboveground weed proportion from our experimental subplots.
**Figure S12:** Comparison of non‐target species proportion of reads (weeds and legacy crops) and the difference between actual species proportion and observed proportion of reads. The point colour corresponds with species.

**Data S1:** men70214‐sup‐0002‐SupportingInformation2.docx.

## Data Availability

Sequencing data for this project is available on NCBI Sequence Read Archive under BioProject PRJNA1370150. Data and R code are available on Dryad (https://doi.org/10.5061/dryad.s7h44j1qp).
