# Supplementary material for: Accounting for Variability Across Years, Depths, and Non‐Target Reads Improves Quantification of Root Community Composition in Plant Mixtures
Source: Mol Ecol Resour. 2026 Oct 16;26(7):e70214. doi: 10.1111/1755-0998.70214 (PMC13673398; doi:10.1111/1755-0998.70214)
Supplement: Supplementary file 1 [file MEN-26-e70214-s001.docx]

**SUPPORTING INFORMATION: Accounting for variability across years, depths, and non-target reads improves quantification of root community composition in plant mixtures**

Emma K. Rice, Jason C. Keagy, Terrence H. Bell, Liana T. Burghardt, Jason P. Kaye, and Carolyn J. Lowry

**METHODS**

Table S1. Rock Springs, PA average temperature (°C) and cumulative precipitation (cm) summarized monthly and for each year's study duration. Data from NRCS National Weather and Climate Center site number 2036.

|  | **2021** | | **2022** | | **2023** | |
| --- | --- | --- | --- | --- | --- | --- |
| **Average** | **Temp (℃)** | **Precip (cm)** | **Temp (℃)** | **Precip (cm)** | **Temp (℃)** | **Precip (cm)** |
| August | 21.80 | 14.02 | 21.16 | 8.84 | 20.18 | 14.33 |
| September | 17.82 | 26.90 | 17.01 | 0.64 | 16.41 | 9.47 |
| October | 13.85 | 8.71 | 9.03 | 2.24 | 11.87 | 7.77 |
| November | 3.65 | 4.85 | 6.18 | 6.58 | 4.44 | 5.18 |
| Aug - Nov | 14.34 | 54.48 | 13.38 | 18.29 | 13.27 | 36.75 |

Table S2. Cover crop focal species abbreviations, planted cultivars, and seeding rates. These seeding rates have been used at the site from 2019 onward.

| **Focal Species** | **Abbreviation** | **Cultivar** | **Seeding Rate (m^2^)** |
| --- | --- | --- | --- |
| Crimson clover | Clover | Dixie | 600 |
| Austrian winter pea | Pea | VNS ^†^ | 60 |
| Canola | Canola | Wichita | 400 |
| Triticale | Triticale | VNS, Trical Grainer 154, Trical Surge^‡^ | 300 |
| † VNS is variety not specified.  ‡ Cultivar names were planted in consecutive years from 2021 to 2023 in the order they are listed. Triticale’s cultivar variety changed each year due to the challenge of finding consistently available organic seeds with good germination. | | | |

Table S3. Dates of field operations and sample processing.

|  | **Year 1 (Fall 2021)** | **Year 2 (Fall 2022)** | **Year 3 (Fall 2023)** |
| --- | --- | --- | --- |
| Planting | 8/26 | 8/26 | 8/29 |
| Weeding | 9/28, 10/15^†^ | 10/7 - 10/18 | 10/11 - 10/26 |
| Root collection | 11/11 - 11/16 | 11/15 - 11/29^‡^ | 11/14 - 11/17 |
| Root washing | 11/23 - 12/15 | 11/30 - 12/19 | 12/1 - 1/13 |

† Weeded all plots twice. In subsequent years, weeded each plot once within the date range.

‡ Frozen soil prolonged root collection.

Table S4. Reference database metadata. If no accession number is listed, the sequence was derived from a local sample and Sanger sequencing.

| **Genus Species** | **Category** | **NCBI Accession** | **Query Coverage (%)** | **Identity (%)** |
| --- | --- | --- | --- | --- |
| *Brassica napus* | focal species |  | 100 | 99.38 |
| *Trifolium incarnatum* | focal species |  | 100 | 99.63 |
| *Pisum sativum* | focal species |  | 99 | 100 |
| *x Triticosecale* | focal species |  | 100 | 100 |
| *Secale cereale* | crop |  | 100 | 100 |
| *Zea mays* | crop |  | 100 | 100 |
| *Glycine max* | crop |  | 100 | 100 |
| *Triticum aestivum* | crop |  | 100 | 100 |
| *Abutilon theophrasti* | weed |  | 100 | 99.59 |
| *Amaranthus hybridus* | weed |  | 100 | 99.79 |
| *Ambrosia artemisiifolia* | weed |  | 100 | 99.76 |
| *Chenopodium album* | weed |  | 99 | 100 |
| *Cirsium arvense* | weed |  | 100 | 100 |
| *Cyperus esculentus* | weed | OM672242.1 | 100 | 100 |
| *Impatiens capensis* | weed |  | 100 | 99.79 |
| *Lamium amplexicaule* | weed | JN891014.1 | 100 | 99.2 |
| *Oxalis corniculata* | weed | MF695000.1 | 100 | 100 |
| *Persicaria pensylvanica* | weed | MK526318.1 | 100 | 99.2 |
| *Plantago major* | weed |  | 100 | 100 |
| *Rumex crispus* | weed |  | 100 | 100 |
| *Salvia farinacea* | weed |  | 100 | 100 |
| *Setaria faberi* | weed |  | 100 | 99.79 |
| *Solanum ptychanthum* | weed | MG224055.1 | 100 | 100 |
| *Stellaria media* | weed |  | 100 | 99.79 |
| *Taraxacum officinale* | weed | MG223282.1 | 100 | 100 |
| *Trifolium repens* | weed | OR822921.1 | 100 | 99.28 |

Table S5. A global model was input into the dredge function from the *MuMin* package in R, from which the best model for each species was selected based on predictive accuracy.

*spp.mix.prop ~ spp.prop.reads + year + depth + treatment +*

*non.target.prop.reads + year:spp.prop.reads +*

*depth:spp.prop.reads + treatment:spp.prop.reads +*

*non.target.prop.reads:spp.prop.reads +*

*year:depth:spp.prop.reads + (1|block)*

Table S6. Simulation levels in calibration simulation to determine how many levels are needed to predict species proportions in simple mixtures accurately. Within each level, all years, depths, and treatments are represented if possible (there were some proportional ranges where we did not have all variables present to model).

| **Levels** | **Proportion Ranges Within Each Level** |
| --- | --- |
| 2 | (0, 0.1), (0.6, 1.0) |
| 3 | (0, 0.1), (0.3, 0.4), (0.6, 1.0) |
| 4 | (0, 0.1), (0.25, 0.35), (0.45, 0.55), (0.6, 1.0) |
| 5 | (0, 0.1), (0.15, 0.25), (0.3, 0.4), (0.45, 0.55), (0.6, 1.0) |
| 6 | (0, 0.1), (0.15, 0.25), (0.25, 0.35), (0.35, 0.45), (0.45, 0.55), (0.6, 1.0) |
| 7 | (0, 0.1), (0.1, 0.2), (0.2, 0.3), (0.3, 0.4), (0.4, 0.5), (0.5, 0.6), (0.6, 1.0) |
| 8 | (0, 0.1), (0.1, 0.15), (0.15, 0.2), (0.25, 0.3), (0.35, 0.4), (0.45, 0.5), (0.55, 0.6), (0.6, 1.0) |
| 9 | (0, 0.1), (0.1, 0.15), (0.15, 0.2), (0.25, 0.3), (0.35, 0.4), (0.45, 0.5), (0.5, 0.55), (0.55, 0.6), (0.6, 1.0) |
| 10 | (0, 0.1), (0.1, 0.15), (0.15, 0.2), (0.2, 0.25), (0.3, 0.35), (0.4, 0.45), (0.45, 0.5), (0.5, 0.55), (0.55, 0.6), (0.6, 1.0) |
| 11 | (0, 0.1), (0.1, 0.15), (0.15, 0.2), (0.2, 0.25), (0.25, 0.3), (0.35, 0.4), (0.4, 0.45), (0.45, 0.5), (0.5, 0.55), (0.55, 0.6), (0.6, 1.0) |

**RESULTS**

Table S7. Base model with only proportion of reads as the explanatory factor and block as a random factor for each species to predict the proportion mass of a species in an unknown mixture. Reported each models associated fit statistics: root mean squared error (RMSE), mean average error (MAE) and R^2^.

| **Species** | **Model** | **RMSE** | **MAE** | **R^2^** |
| --- | --- | --- | --- | --- |
| Canola | canola.prop.mix ~ canola.prop.reads + (1\|block) | 0.19 | 0.15 | 0.68 |
| Clover | clover.prop.mix ~ clover.prop.reads + (1\|block) | 0.20 | 0.15 | 0.60 |
| Pea | pea.prop.mix ~ pea.prop.reads + (1\|block) | 0.24 | 0.20 | 0.37 |
| Triticale | trtitcale.prop.mix ~ triticale.prop.reads + (1\|block) | 0.25 | 0.21 | 0.36 |

Figure S1. The relationship between the actual species mass proportion in the calibration mixture and proportion of sequencing reads. The point color corresponds with species; the solid gray line is the target relationship with a slope of one and an intercept of zero.

Figure S2. The relationship between actual canola mass proportion and predicted canola mass proportion of all samples across years in mixture when calibrating by individual years and all years (calibration year labeled on each plot). The solid gray line is the target relationship with a slope of one and intercept of zero; the dashed black line is the fitted linear model.

Figure S3. The relationship between actual clover mass proportion and predicted clover mass proportion in mixture when calibrating by individual years and all years (calibration year labeled on each plot). The solid gray line is the target relationship with a slope of one and intercept of zero; the dashed black line is the fitted linear model.

Figure S4. The relationship between actual pea mass proportion and predicted pea mass proportion in mixture when calibrating by individual years and all years (calibration year labeled on each plot). The solid gray line is the target relationship with a slope of one and intercept of zero; the dashed black line is the fitted linear model.

Figure S5. The relationship between actual triticale mass proportion and predicted triticale mass proportion in mixture when calibrating by individual years and all years (calibration year labeled on each plot). The solid gray line is the target relationship with a slope of one and intercept of zero; the dashed black line is the fitted linear model.

Figure S6. Relationship between actual canola mass proportion and predicted canola mass proportion when calibrating by individual depth segments and all depths (calibration depth labeled on each plot). The solid gray line is the target relationship with a slope of one and intercept of zero, the dashed black line is the fit linear model.

Figure S7. Relationship between actual clover mass proportion and predicted clover mass proportion when calibrating by individual depth segments and all depths (calibration depth labeled on each plot). The solid gray line is the target relationship with a slope of one and intercept of zero, the dashed black line is the fit linear model.

Figure S8. Relationship between actual pea mass proportion and predicted pea mass proportion when calibrating by individual depth segments and all depths (calibration depth labeled on each plot). The solid gray line is the target relationship with a slope of one and intercept of zero, the dashed black line is the fit linear model.

Figure S9. Relationship between actual triticale mass proportion and predicted triticale mass proportion when calibrating by individual depth segments and all depths (calibration depth labeled on each plot). The solid gray line is the target relationship with a slope of one and intercept of zero, the dashed black line is the fit linear model.

Figure S10. Simulation of random calibration mixtures to verify that model simulations are better than random chance when using a large dataset. Simulation includes 500 replicates to determine a) how many reads per sample are needed to attain a high R^2^ and c) low root mean squared error (RMSE) and 10,000 replicates to determine b) how many calibration levels are needed for a high R^2^ and d) low RMSE. The point and line color corresponds with the species. Simulated reads per sample experiment went up to 35,000, but we are only showing up to 20,000 reads here.

Figure S11. Comparison of nontarget species proportion of reads (weeds and legacy crops) and the aboveground weed proportion from our experimental subplots.

Figure S12. Comparison of non-target species proportion of reads (weeds and legacy crops) and the difference between actual species proportion and observed proportion of reads. The point color corresponds with species.
