# Supplementary material for: Accounting for Variability Across Years, Depths, and Non‐Target Reads Improves Quantification of Root Community Composition in Plant Mixtures
Source: Mol Ecol Resour. 2026 Oct 16;26(7):e70214. doi: 10.1111/1755-0998.70214 (PMC13673398; doi:10.1111/1755-0998.70214)
Supplement: Supplementary file 2 [file MEN-26-e70214-s002.docx]

**REFERENCE DATABASE: Accounting for variability across years, depths, and non-target reads improves quantification of root community composition in plant mixtures**

Emma K. Rice, Jason C. Keagy, Terrence H. Bell, Liana T. Burghardt, Jason P. Kaye, and Carolyn J. Lowry

>Plantae;Tracheophyta;Magnoliopsida;Poales;Poaceae;Triticum;aestivum

GATGCTATCACATCGAGCCTGTTGCTGGGGAAGACAGCCAATGGATCTGT

TATGTAGCTTATCCATTAGACCTATTTGAAGAGGGTTCCGTTACTAACAT

GTTTACTTCCATTGTAGGTAACGTATTTGGTTTCAAAGCCCTACGTGCTC

TACGTTTGGAGGATCTACGAATTCCCCCTACTTATTCAAAAACTTTCCAA

GGCCCGCCTCATGGTATCCAAGTTGAAAGAGATAAGTTGAACAAGTATGG

TCGTCCTTTATTGGGATGTACTATTAAACCAAAATTGGGATTATCCGCAA

AAAATTATGGTAGAGCGTGTTATGAGTGTCTACGTGGTGGACTTGATTTT

ACCAAAGATGATGAAAACGTAAACTCACAACCATTTATGCGCTGGAGAGA

CCGTTTTGTCTTTTGTGCCGAAGCTATTTATAAATCACAGGCCGAAACCG

GTGAAATCAAGGGGCATTACTTGAATGCTACTG

>Plantae;Tracheophyta;Magnoliopsida;Poales;Poaceae;Secale;cereale

GATGCTATCACATCGAGCCTGTTGCTGGGGAAGACAACCAATGGATCTGT

TATGTAGCTTATCCATTAGACCTATTTGAAGAGGGTTCCGTTACTAACAT

GTTTACTTCCATTGTGGGTAACGTATTTGGTTTCAAAGCCCTACGTGCTC

TACGTTTGGAGGATCTACGAATTCCCCCTACTTATTCAAAAACTTTCCAA

GGCCCGCCTCATGGTATCCAAGTTGAAAGAGATAAGTTGAACAAGTATGG

TCGTCCTTTATTGGGATGTACTATTAAACCAAAATTGGGATTATCCGCAA

AAAATTATGGTAGAGCGTGTTATGAGTGTCTACGTGGTGGACTTGATTTT

ACCAAAGATGATGAAAACGTAAACTCACAACCATTTATGCGCTGGAGAGA

CCGTTTTGTCTTTTGTGCCGAAGCTATTTATAAATCACAGGCCGAAACCG

GTGAAATCAAGGGGCATTACTTGAATGCTACTGCA

>Plantae;Magnoliophyta;Magnoliopsida;Caryophyllales;Caryophyllaceae;Stellaria;media

CATCGAGCCCGTTGCTGGAGAAGAAAATCAATATATTTGTTATGTAGCTT

ACCCCTTAGACCTTTTTGAGGAAGGCTCTGTTACTAACATGTTTACTTCC

ATTGTGGGTAATGTATTTGGGTTCAAAGCCTTGCGTGCTCTACGCTTGGA

AGATTTGCGAATTCCTGTTGCTTATATAAAAACTTTCCAAGGCCCGCCTC

ACGGTATCCAAGTTGAAAGAGATAAATTGAACAAGTATGGCCGTCCCCTA

TTGGGATGCACCATTAAACCGAAATTAGGGTTATCCGCTAAAAACTATGG

TCGCGCAGTTTATGAATGTCTTCGTGGTGGACTTGATTTTACCAAAGATG

ATGAAAACGTGAACTCTCAACCATTTATGCGCTGGAGAGACCGCTTCTTA

TTTTGTGCCGAAGCCATTTATAAAGCACAAGCCGAAACAGGTGAAATCAA

AGGGCATTACTTGAATGCTACTG

>Plantae;Anthophyta;Monocotyledoneae;Cyperales;Poaceae;Zea;mays

GATGCTATCACATCGAGCCCGTTCCTGGGGACCCAGATCAATATATCTGT

TATGTAGCTTATCCATTAGACCTATTTGAAGAGGGTTCTGTTACTAACAT

GTTTACTTCCATTGTGGGTAACGTATTTGGTTTCAAAGCCTTACGCGCTC

TACGTTTGGAGGATCTACGAATTCCCCCTGCTTATTCAAAAACTTTCCAA

GGTCCGCCTCACGGTATCCAAGTTGAAAGGGATAAGTTGAACAAGTACGG

TCGTCCTTTATTGGGATGTACTATTAAACCAAAATTGGGATTATCCGCAA

AAAATTACGGTAGAGCGTGTTATGAGTGTCTACGCGGTGGACTTGATTTT

ACCAAAGATGATGAAAACGTAAACTCACAACCATTTATGCGCTGGAGAGA

CCGTTTTGTCTTTTGTGCCGAAGCAATTTATAAAGCACAAGCCGAAACTG

GTGAAATCAAGGGGCATTACTTGAATGCTACTG

>Plantae;Magnoliophyta;Magnoliopsida;Asterales;Asteraceae;Cirsium;arvense

CCATTAGACCTTTTTGAAGAAGGTTCTGTTACTAACATGTTTACTTCCAT

TGTAGGTAATGTATTTGGGTTCAAAGCCCTGCGTGCTCTACGTCTGGAAG

ATTTGCGAATCCCTACTGCGTATGTTAAAACTTTCCAAGGTCCGCCTCAC

GGCATCCAAGTTGAGAGAGATAAATTGAACAAGTATGGTCGTCCCCTGTT

GGGATGTACTATTAAACCTAAATTGGGGTTATCCGCTAAAAACTATGGTA

GAGCTGTTTATGAATGTCTTCGTGGTGGCCTTGATTTTACTAAAGATGAT

GAGAACGTGAACTCCCAACCATTTATGCGTTGGAGAGACCGTTTCTTATT

TTGTGCCGAAGCTCTTTATAAAGCACAAGCTGAAACAGGTGAAATCAAAG

GGCATTACTTGA

>Plantae;Magnoliophyta;Magnoliopsida;Malvales;Malvaceae;Abutilon;theophrasti

GATGCTACCACATTGAGCCCGTTGCTGGAGAAGAAGATCAATATATATGT

TATGTAGCTTACCCCTTAGACCTTTTTGAAGAAGGTTCTGTTACTAACAT

GTTTACTTCCATTGTGGGTAATGTATTTGGGTTCAAAGCCCTGCGCGCTC

TACGTTTAGAAGATCTGCGAATCCCTATTTCTTATATTAAAACTTTCCAA

GGCCCGCCTCATGGCATCCAGGTTGAAAGAGATAAATTGAACAAGTATGG

TCGCCCCCTATTAGGATGTACTATTAAACCTAAATTGGGGTTATCCGCTA

AGAACTACGGTAGAGCAGTTTATGAATGTCTACGTGGTGGACTTGATTTT

ACCAAAGATGATGAGAATGTGAACTCCCAACCATTTATGCGCTGGAGAGA

CCGTTTCTTATTTTGTGCCGAAGCACTTTATAAAGCACAGGCTGAAACAG

GTGAAATCAAGGGGCATTACTTGAATGCTACTGCA

>Plantae;Spermatophyta;Monocotyledonae;Cyperales;Poaceae;Setaria;faberi

TCACATCGAGCCCGTTCCTGGGGAGGCAGATCAATATATCTGTTATATAG

CTTATCCATTAGACCTATTTGAAGAGGGTTCTGTTACTAACATGTTTACT

TCCATTGTGGGTAACGTGTTTGGTTTCAAAGCCCTACGCGCTCTACGTTT

GGAGGATCTACGAATTCCCACTGCTTATTCAAAAACTTTCCAAGGTCCGC

CTCACGGTATCCAAGTTGAAAGGGATAAGTTGAACAAGTATGGTCGTCCT

TTATTGGGATGTACTATTAAACCAAAATTGGGATTATCCGCAAAAAATTA

CGGTAGAGCGTGTTATGAGTGTCTACGCGGTGGACTTGATTTTACCAAAG

ATGATGAAAACGTAAACTCACAACCATTTATGCGCTGGAGAGACCGTTTT

GTCTTTTGTGCTGAAGCAATTTATAAAGCACAAGCAGAAACTGGTGAAAT

TAAGGGGCATTACTTGAATGCTACTG

>Plantae;Magnoliophyta;Magnoliopsida;Fabales;Fabaceae;Glycine;max

TGTTGCTGGGGAAGAAAATCAATATATTGCTTATGTAGCTTATCCCTTAG

ACCTTTTTGAAGAAGGTTCTGTTACTAACATGTTTACTTCCATTGTCGGT

AATGTATTTGGGTTCAAGGCCCTGCGTGCTCTACGTCTGGAGGATTTGCG

AATCCCTACTGCTTATATTAAAACTTTCCAAGGTCCGCCTCATGGCATCC

AAGTTGAGAGAGATAAATTGAACAAGTATGGTCGTCCCCTATTAGGATGT

ACTATTAAACCTAAATTGGGGTTATCCGCTAAGAATTATGGTAGAGCTGT

TTATGAATGTCTTCGTGGGGGACTTGATTTTACCAAAGATGATGAAAATG

TGAATTCCCAACCATTTATGCGTTGGAGAGACCGTTTCTTATTTTGTGCC

GAAGCCATTTTTAAATCACAGGCTGAAACAGGTGAAATCAAAGGGCATTA

CTTGAATGCTACTGCA

>Plantae;Streptophyta;Equisetopsida;Caryophyllales;Amaranthaceae;Chenopodium;album

ATGCTACCACATCGAGCCTGTTGCTGGAGAAGAAAATCAATATATTTGTT

ATGTAGCGTATCCCTTAGACCTTTTTGAAGAAGGTTCTGTTACTAACATG

TTTACTTCCATTGTGGGTAACGTATTTGGGTTCAAAGCTCTGCGTGCTCT

ACGTTTGGAGGATTTACGAATCCCTGTTGCTTATATAAAAACTTTCCAAG

GCCCGCCTCACGGTATCCAAGTTGAGAGAGATAAATTGAACAAGTACGGC

CGCCCCCTATTGGGATGCACTATTAAACCAAAATTGGGGTTATCCGCTAA

AAACTATGGTCGAGCATGTTATGAATGTCTTCGTGGTGGACTTGATTTTA

CCAAAGATGATGAAAACGTGAACTCCCAGCCGTTTATGCGTTGGAGAGAC

CGTTTCCTATTTTGTGCCGAAGCTATTTATAAAGCACAAGCCGAAACAGG

CGAAATCAAAGGTCATTACTTGAATGCTACTGCAGG

>Plantae;Anthophyta;Dicotyledoneae;Asterales;Asteraceae;Taraxacum;officinale

AGTGTTGGATTCAAAGCTGGTGTTAAAGATTATAAATTGACTTATTATAC

TCCTGACTATGAAACCAAGGATACTGATATTTTGGCAGCATTTCGAGTAA

CTCCTCAACCTGGAGTTCCGCCTGAAGAAGCAGGGGCCGCAGTAGCTGCC

GAATCTTCTACTGGTACATGGACAACTGTGTGGACCGATGGACTTACGAG

CCTTGATCGTTACAAAGGGCGATGCTATGGAATTGAGCCTGTTCCTGGAG

AAGAAAGTCAATTTATTGCTTATGTAGCTTACCCATTAGACCTTTTTGAA

GAAGGTTCTGTTACTAACATGTTTACTTCCATTGTAGGTAATGTATTTGG

GTTCAAAGCCCTGCGTGCTCTACGTCTGGAAGATTTGCGAATCCCTGTTG

CGTATGTTAAAACTTTCCAAGGTCCGCCTCACGGCATCCAAGTTGAGAGA

GATAAATTGAACAAGTATGGTCGTCCTCTGTTGGGATGTACTATTAAACC

TAAATTGGGGTTATCCGCTAAAAACTACGGTAGAGCTGTTTATGAATGTC

TT

>Plantae;Magnoliophyta;Magnoliopsida;Plantaginales;Plantaginaceae;Plantago;major

CGATGCTACCACATTGAGCCCGTTCCTGGAGAAGCAGATCAATATATCTG

TTATGTAGCTTACCCTTTAGACCTTTTTGAAGAAGGGTCTGTTACTAACA

TGTTTACTTCCATTGTAGGAAATGTATTTGGATTCAAAGCCCTGCGTGCT

CTACGTCTGGAAGATCTACGAATCCCTGTTGCTTATGTTAAAACTTTCCA

AGGCCCGCCTCACGGGATCCAAGTTGAGAGAGATAAATTGAACAAGTACG

GTCGTCCCCTGTTGGGATGTACTATTAAACCTAAATTGGGGTTATCTGCT

AAAAACTATGGTAGAGCATGTTATGAATGTCTTCGTGGTGGACTTGATTT

TACCAAAGATGATGAGAACGTGAACTCCCAGCCATTTATGCGTTGGAGAG

ACCGTTTCTTATTCTGTGCCGAAGCTATTTATAAATCACAGGCGGAAACA

GGTGAAATCAAAGGGCATTACTTAAATGCTACTG

>Plantae;Spermatophyta;Dicotyledonae;Asterales;Asteraceae;Ambrosia;artemisiifolia

CCACTACACCTTTTTGAAGAAGGTTCTGTTACTAACATGTTTACTTCCAT

TGTAGGTAATGTATTTGGGTTCAAAGCCCTGCGTGCTCTACGTCTGGAAG

ATTTGCGAATCCCGACTGCGTATGTTAAAACTTTCGACGGTCCGCCTCAC

GGTATCCAAGTTGAGAGAGATAAATTGAACAAGTATGGTCGTCCCCTGTT

GGGATGTACTATTAAACCTAAATTGGGGTTATCCGCTAAAAACTACGGTA

GAGCTTGTTATGAATGTCTTCGTGGTGGCCTTGACTTTACTAAAGATGAT

GAGAACGTGAACTCCCAACCATTTATGCGTTGGAGAGACCGTTTCTTATT

TTGTGCCGAAGCTATTTATAAAGCACAAGCTGAAACAGGTGAAATCAAAG

GGCATTACTTGAA

>Plantae;Spermatophyta;Magnoliopsida;Caryophyllales;Amaranthaceae;Amaranthus;hybridus

ACATCGAGCCCGTTGCTGGAGAAGAAAATCAATATATTTGTTATGTAGCG

TATCCTTTAGACCTTTTTGAAGAAGGTTCTGTTACTAACATGTTTACTTC

CATTGTGGGTAACGTATTTGGGTTCAAAGCTTTGCGTGCTCTACGTTTGG

AAGATTTGCGAATCCCTGTTGCTTATGTCAAAACTTTCCAAGGCCCGCCT

CACGGTATCCAGGTTGAAAGAGATAAATTGAACAAGTACGGTCGTCCCCT

ATTGGGATGCACTATTAAACCAAAATTGGGGTTATCCGCTAAAAACTATG

GTCGAGCATGTTATGAATGTCTTCGTGGTGGACTTGATTTTACCAAAGAT

GATGAAAACGTGAATTCCCAGCCGTTCATGCGTTGGAGAGACCGTTTCCT

ATTTTGTGCCGAAGCTATTTATAAATCACAAGCCGAAACAGGTGAAATCA

AAGGGCATTATTTGAATGCTACTGCA

>Plantae;Magnoliophyta;Magnoliopsida;Polygonales;Polygonaceae;Rumex;crispus

CATCGAGCCTGTTCCTGGAGAAGAAAGTCAGTTTATTGCTTATGTAGCTT

ACCCATTAGACCTTTTTGAAGAAGGTTCTGTTACTAACATGTTTACTTCC

ATTGTGGGTAATGTATTTGGGTTCAAAGCCCTGCGTGCTCTACGTTTGGA

GGATTTGCGAATCCCTCCTGCTTATACGAAAACTTTCCAAGGCCCGCCTC

ATGGTATCCAAGTTGAAAGAGATAAATTGAACAAATATGGACGTCCCCTA

TTGGGATGTACTATTAAACCGAAATTGGGGTTGTCCGCTAAGAACTACGG

CCGAGCAGTTTATGAATGTCTTCGTGGCGGACTTGATTTTACCAAAGATG

ATGAAAACGTGAACTCCCAACCATTTATGCGTTGGAGAGACCGTTTCTTA

TTCTGTGCCGAAGCTATTTTTAAATCACAGTCTGAAACAGGTGAAATCAA

AGGACATTACTTAAATGCTACTGCAGG

>Plantae;Tracheophytes;Asterids;Lamiales;Lamiaceae;Salvia;farinacea

CGATGCTACCACATTGAGCCCGTTCCTGGAGAAAAAGATCAATATATCTG

TTATGTAGCTTACCCTTTAGACCTTTTTGAAGAAGGTTCTGTTACTAACA

TGTTTACTTCCATTGTAGGAAATGTATTTGGATTCAAAGCTCTACGTGCT

CTACGTCTGGAAGATCTGCGAATTCCTGTTGCTTATGTTAAAACTTTCCA

AGGCCCGCCTCATGGGATCCAAGCTGAGAGAGATAAATTGAACAAGTACG

GTCGTCCTCTGCTTGGATGTACTATTAAACCTAAATTGGGGTTATCTGCT

AAAAACTATGGTAGAGCGGTTTATGAATGTCTTCGCGGTGGACTTGATTT

TACCAAAGATGATGAGAACGTGAACTCCCAGCCATTTATGCGTTGGAGAG

ACCGCTTCTTATTTTGTGCCGAAGCAATTTATAAATCACAGGCTGAAACA

GGTGAAATCAAAGGGCATTACTTGAATGCTACTG

>Plantae;Magnoliophyta;Magnoliopsida;Geraniales;Balsaminaceae;Impatiens;capensis

GATGCTATCACATCGAGCCAGTTGCTGGAGAAGAAAATCAATTTATTGCT

TATGTAGCTTACCCTTTAGACCTTTTTGAAGAAGGTTCTGTTACTAACAT

GTTTACTTCCATTGTGGGTAATGTATTTGGGTTCAAAGCCCTGCGCGCTC

TACGTCTGGAAGATCTTCGAATCCCTCCTGCGTATACTAAAACTTTCCAA

GGACCGCCTCATGGCATCCAAGTTGAGAGAGATAAGTTGAACAAGTATGG

TCGTCCCCTGTTGGGATGTACTATTAAACCTAAATTGGGGTTATCCGCTA

AAAACTATGGTAGAGCAGTTTATGAATGTCTCCGCGGTGGACTTGATTTT

ACCAAAGATGATGAGAACGTGAACTCTCAACCATTTATGCGTTGGCGAGA

CCGTTTCTTGTTTTGTGCCGAAGCAATTTATAAATCACAGGCCGAAACAG

GTGAAATCAAAGGGCATTACTTAAATGCTACTGCAGG

>Plantae;Tracheophyta;Magnoliopsida;Fabales;Fabaceae;Trifolium;incarnatum

TGTTAAGATTATAGGTTGACTTATTATACTCCTGACTATGAAACCAAAGA

TACTGATATCTTGGCAGCATTCCGAGTAACTCCTCAACCCGGAGTTCCAC

CTGAAGAAGCAGGTGCAGCGGTAGCTGCCGAATCTTCCACTGGGACATGG

ACAACTGTGTGGACCGATGGACTTACCAGTCTTGATCGTTATAAAGGACG

CTGCTATCACATCGAGCCTGTTGCTGGAGAAGATACTCAATTTATTGCTT

ATGTAGCTTATCCCTTAGACCTTTTTGAAGAAGGTTCTGTTACTAACATG

TTTACCTCTATTGTAGGTAATGTATTTGGGTTCAAGGCCTTGCGTGCTCT

ACGTCTGGAAGATTTGCGAATCCCCGTTGCTTATGTTAAAACTTTCCAAG

GTCCTCCTCACGGAATCCAAGTTGAGAGAGATAAATTGAACAAGTATGGA

CGTCCCCTATTGGGATGTACTATTAAACCTAAATTGGGTTTATCCGCTAA

GAATTACGGTAGAGCAGTTTATGAATGTCTACGCGGTG

>Plantae;Tracheophyta;Magnoliopsida;Fabales;Fabaceae;Pisum;sativum

TATACTCCTGACTATCAAACCAAAGATACTGATATCTTGGCAGCATTCCG

AGTAACTCCTCAACCTGGAGTTCCGCCTGAAGAAGCAGGTGCAGCGGTAG

CTGCAGAATCTTCCACTGGTACATGGACAACTGTGTGGACCGATGGACTT

ACGAGCCTCGATCGTTATAAAGGACGCTGCTACGAGATCGAGCCTGTTCC

TGGAGAAGATAATCAATTTATTGCTTATGTAGCTTATCCCTTAGACCTTT

TTGAAGAAGGTTCTGTTACTAACATGTTTACCTCCATTGTAGGTAATGTA

TTTGGGTTCAAGGCCTTGCGTGCTCTACGTCTGGAAGATTTGCGAATCCC

TTATGCTTATGTTAAAACTTTCCAAGGTCCTCCTCACGGAATCCAAGTTG

AGAGAGATAAATTGAACAAGTATGGACGTCCCCTATTGGGATGTACTATT

AAACCAAAATTGGGTTTATCCGCTAAGAATTATGGTAGAGCAGTTTATGA

ATGTCTCCGCGGTGGCT

>Plantae;Tracheophyta;Magnoliopsida;Poales;Poaceae;x_Triticosecale;x_Triticosecale_Wittmack

GTTAAGATTATAAATTGACTTACTACACCCCAGAGTATGAAACTAAGGAT

ACTGATATCTTGGCAGCATTCCGAGTAAGTCCTCAGCCTGGGGTTCCGCC

CGAAGAAGCAGGGGCTGCAGTAGCTGCCGAATCTTCTACTGGTACATGGA

CAACTGTTTGGACTGATGGACTTACCAGTCTTGATCGTTACAAAGGACGA

TGCTATCACATCGAGCCTGTTGCTGGGGAAGACAGCCAATGGATCTGTTA

TGTAGCTTATCCATTAGACCTATTTGAAGAGGGTTCCGTTACTAACATGT

TTACTTCCATTGTAGGTAACGTATTTGGTTTCAAAGCCCTACGTGCTCTA

CGTTTGGAGGATCTACGAATTCCCCCTACTTATTCAAAAACTTTCCAAGG

CCCGCCTCATGGTATCCAAGTTGAAAGAGATAAGTTGAACAAGTATGGTC

GTCCTTTATTGGGATGTACTATTAAACCAAAATTGGGATTATCCGCAAAA

AATTATGGTAGAGCGTGTTATGAGTGTCTACGCGGTG

>Plantae;Tracheophyta;Magnoliopsida;Brassicales;Brassicaceae;Brassica;napus

ATGCTACCACATCGAGCCCGTTCCAGGAGAAGAAACTCAATTTATTGCTG

TATGTAGCTTACCCTTTAGACCTTTTTGAAGAAGGGTCTGTTACTAACAT

GTTTACCTCAATTGTGGGTAATGTATTTGGGTTCAAAGCCCTGGCTGCTC

TACGTCTAGAGGATCTGCGAATCCCTCCGGCTTATACTAAAACTTTCCAG

GGACCACCTCATGGTATCCAAGTTGAAAGAGATAAATTGAACAAGTATGG

ACGTCCCCTATTAGGATGTACTATTAAACCTAAGTTGGGGTTATCCGCGA

AGAACTATGGTAGAGCAGTTTATGAATGTCTACGTGGTGGACTTGATTTT

ACCAAAGATGATGAGAATGTGAACTCTCAACCATTTATGCGTTGGAGAGA

CCGTTTCTTATTTTGTGCCGAAGCTATTTATAAATCACAGGCTGAAACAG

GTGAAATCAAAGGACATTATTTGAATGCTACTG

>Plantae;Magnoliophyta;Magnoliopsida;Solanales;Solanaceae;Solanum;ptychanthum

AGTGTTGGATTCAAAGCTGGTGTTAAAGAGTACAAATTGACTTATTATAC

TCCTGAGTACCAAACCAAGGATACTGATATATTGGCAGCATTCCGAGTAA

CTCCTCAACCTGGAGTTCCACCTGAAGAAGCAGGGGCCGCGGTAGCTGCC

GAATCTTCTACTGGTACATGGACAACTGTATGGACCGATGGACTTACCAG

TCTTGATCGTTACAAAGGGCGATGCTACCGCATCGAGCGTGTTGTTGGAG

AAAAAGATCAATATATTGCTTATGTAGCTTACCCTTTAGACCTTTTTGAA

GAAGGTTCCGTTACCAATATGTTTACTTCCATTGTAGGTAATGTATTTGG

GTTCAAAGCCCTGCGCGCTCTACGTCTGGAAGATCTGCGAATCCCTACTG

CTTATGTTAAAACTTTCCAAGGTCCGCCTCATGGGATCCAAGTTGAAAGA

GATAAATTGAACAAGTATGGTCGTCCCCTGTTGGGATGTACTATTAAACC

TAAATTGGGGTTATCTGCTAAAAACTATGGTAGAGCTGTTTATGAATGTC

TT

>Plantae;Magnoliophyta;Magnoliopsida;Geraniales;Oxalidaceae;Oxalis;corniculata

AAGTGTTGGATTCAAGGCCGGTGTTAAAGATTATAAATTGACTTATTATA

CTCCTGAGTATGAAACCAAAGATACTGATATCTTGGCAGCATTCCGAGTA

ACTCCTCAACCTGGAGTTCCTCCTGAGGAAGCTGGGGCAGCGGTAGCTGC

TGAATCTTCTACTGGTACATGGACAACTGTGTGGACCGATGGGCTTACCA

GTCTTGATCGTTACAAAGGACGATGCTACCACATCGAGCCTGTTCCTGGA

GAAGAAAGTCAATTTATTGCTTATGTAGCTTACCCCTTAGACCTTTTTGA

AGAAGGTTCTGTTACTAACATGTTTACTTCCATTGTGGGTAATGTGTTTG

GGTTCAAGGCCCTGCGGGCTCTACGTTTGGAGGATTTGCGAATCCCTCCT

GCTTATACTAAAACTTTTCAAGGCCCGCCTCATGGTATTCAGGTTGAGAG

AGATAAGTTGAACAAGTACGGCCGCCCCCTATTAGGATGTACTATTAAAC

CTAAATTGGGGTTATCCGCTAAGAATTACGGTAGAGCAGTTTATGAATGT

CTTC

>Plantae;Magnoliophyta;Liliopsida;Cyperales;Cyperaceae;Cyperus;esculentus

CTTACTTATTATACTCCTGAGTACGAAACCAAAGATACTGATATCTTGGC

AGCGTTCCGAGTAACTCCTCAACCTGGAGTCCCTCCTGAAGAAGCAGGAG

CTGCAGTAGCGGCGGAATCTTCTACTGGTACATGGACAACTGTTTGGACT

GATGGACTTACCAGTCTTGATCGTTACAAAGGGCGATGCTATCATATCGA

ACCTGTTGCTGGAGAAGAAAATCAATATATTGCCTATATAGCTTATCCTT

TAGACCTTTTCGAAGAAGGTTCTGTTACTAACATGTTTACTTCTATTGTA

GGTAATGTATTTGGTTTCAAAGCCTTACGAGCTCTACGCTTGGAAGACTT

ACGAATTCCTCCTGCTTATTCAAAAACTTTCCAAGGTCCACCTCACGGTA

TCCAAGCTGAAAGAGATAAGTTGAACAAGTATGGTCGTCCTCTATTGGGA

TGTACTATTA

>Plantae;Magnoliophyta;Magnoliopsida;Fabales;Fabaceae;Trifolium;repens

AAAGGTTGGGTTCAAAGCTGGTGTTAAAGATTATAGGTTGACTTATTATA

CTCCTGACTATGAAACCAAAGATACCGATATCTTGGCAGCATTCCGAGTA

ACTCCTCAACCCGGAGTTCCGCCTGAAGAAGCAGGTGCAGCGGTAGCTGC

CGAATCTTCCACTGGGACATGGACAACTGTGTGGACCGATGGACTTACCA

GTCTTGATCGTTATAAAGGACGCTGCTACCACATCGAGCCGGTTGCTGGA

GAAGAAAGTCAATTTATTGCTTATGTAGCTTATCCCTTAGACCTTTTTGA

AGAAGGTTCTGTTACTAACATGTTTACCTCCATTGTAGGTAATGTATTTG

GGTTCAAGGCCTTGCGTGCTCTACGCCTGGAAGATTTGCGAATCCCCGTT

GCTTATGTTAAAACTTTCCAAGGTCCTCCTCACGGAATCCAAGTTGAGAG

AGATAAATTGAACAAGTATGGACGTCCCCTATTGGGATGTACTATTAAAC

CTAAATTGGGTTTATCCGCTAAGAATTACGGTAGAGCAGTTTATGAATGT

CTA

>Plantae;Tracheophyta;Magnoliopsida;Polygonales;Polygonaceae;Persicaria;pensylvanica

AAAGCAAGTGTTGGATTCAAAGCTGGTGTTAAAGAGTATAAATTGAATTA

TTATACTCCTGACTATGAACCCCACGAACATGATATCTTGGCAGCATTTC

GAGTAACTCCTCAACCTGGAGTTCCACCAGAAGAAGCAGGGGCCGCGGTA

GCTGCCGAATCTTCTACTGGTACATGGACAACTGTGTGGACCGATGGACT

TACCAGCCTTGATCGTTACAAAGGACGATGCTACGGCATCGAGCCTGTTG

CTGGAGAAGAAAATCAATATATTGCTTATGTAGCTTACCCATTAGACCTT

TTTGAAGAAGGTTCTGTTACTAACATGTTTACTTCCATTGTAGGTAATGT

ATTTGGGTTCAAAGCCCTGCGTGCTCTACGTTTGGAGGATTTGCGAATCC

CTCCTGCTTATACGAAAACTTTCCAAGGCCCACCTCACGGTATCCAAGTT

GAGAGAGACAAATTAAACAAATACGGACGTCCCCTATTGGGATGTACTAT

TAAACCTAAATTGGGATTGTCCGCTAAGAACTACGGTCGAGCAGTTNNT

>Plantae;Magnoliophyta;Magnoliopsida;Lamiales;Lamiaceae;Lamium;amplexicaule

AGGTGTTGGATTCAAAGCGGGTGTTAAAGAGTACAAATTGACTTATTATAC

CCCTGAATACGAAACCAAAGATACTGATATCTTGGCAGCATTCCGAGTAAC

TCCTCAACCTGGAGTTCCGCCCGAAGAAGCAGGGGCCGCGGTAGCTGCCGA

ATCTTCGACTGGTACATGGACAACTGTGTGGACCGATGGACTTACCAGCCT

TGATCGTTACAAAGGGCGATGCTACGACATCGAGCCCGTTCTTGGAGAAAA

GGATCAATATATCTGTTATGTAGCTTACCCTTTAGACCTTTTTGAAGAAGG

TTCTGTTACTAACATGTTTACTTCGATTGTAGGAAATGTATTTGGATTCAA

AGCCCTACGTGCTCTACGTCTGGAAGATCTGCGAATCCCTCCTGCTTATGT

TAAAACTTTCCAAGGCCCACCTCATGGGATCCAAGTTGAGAGAGATAAATT

GAACAAGTATGGTCGTCCTCTGTTGGGATGTACTATTAAACCTAAATTGGG

GTTATCCGCTAAAAACTATGGTAGAGCTGTTT
